# Ultimate tuning of hyperbolic phonon polaritons

**DOI:** 10.1126/sciadv.adz6278

**Published:** 2025-12-12

**Authors:** Linglong Zhang, Xiaojunjie Ding, Jiahao Dong, Jiang Fan, Wei Wu, Mengxin Ren, Weiwei Luo, Wei Cai, Jingjun Xu

**Affiliations:** ^1^ The Key Laboratory of Weak-Light Nonlinear Photonics, Ministry of Education, School of Physics and TEDA Applied Physics Institute, Nankai University, Tianjin 300457, China.; ^2^ Collaborative Innovation Center of Extreme Optics, Shanxi University, Taiyuan, Shanxi 030006, China.

## Abstract

Volume-confined hyperbolic phonon polaritons (v-HPhPs), which are waveguide modes tightly bounded within anisotropic thin films, represent a distinctive platform for directional manipulations of light-matter interaction at the nanoscale. While the electrically tunable plasmons of atomically thin graphene offers a vital pathway for dynamical control of v-HPhPs in films of hundreds of nanometer thick, achieving the ultimate tuning efficiency is crucial for advancing v-HPhPs related applications. Here, we theoretically establish the fundamental bounds of v-HPhP tuning and experimentally attain this ultimate limit using interlayer-biased double-layer graphene integrated at the v-HPhP film/air interface. This approach is validated through electrical modulation of canalized v-HPhP propagation in α-MoO_3_. Furthermore, we demonstrate the dynamic steering of in-plane directional energy flow at deep-subwavelength scales within mid-infrared range. This breakthrough unlocks promising avenues for dynamic on-chip functionalities, including reconfigurable thermal management and ultrasensitive biosensing.

## Teaser

Interlayer-bias double-layer graphene unlocks ultimate tuning of hyperbolic phonon polaritons.

## INTRODUCTION

Phonon polaritons (PhPs), hybrid quasiparticles arising from photon-phonon coupling, offer advantages for nanophotonics due to their deep subwavelength confinement and low-loss propagation (*1*–*3*). Recently, hyperbolic PhPs (HPhPs) supported in natural materials with low lattice symmetries (*4*–*6*) are emerging as a distinct platform for directional manipulation of light-matter interaction at the nanoscale. The field has evolved from the finding of out-of-plane HPhPs in uniaxial crystals such as hBN (*1*, *7*) to in-plane HPhPs in biaxial materials such as α-MoO_3_ (*8*, *9*) to, most recently, HPhPs in nonhyperbolic crystals (*10*). Reducing lattice symmetry has further uncovered exotic modes, including ghost (*11*) and shear HPhPs (*12*). Crucially, manipulating volume-confined HPhPs (v-HPhPs)–polariton waveguide modes bounded within the films (*1*, *7*–*9*, *13*, *14*) through thickness, interlayer twisting, or dielectric environment engineering, has enabled phenomena such as topological transitions of iso-frequency contours (IFCs) (*14*–*18*) and negative reflection/ refraction (*19*–*22*). Concurrently, advantages of v-HPhPs, such as vibrational strong coupling (*23*), multimodal enhanced quality factor (*24*), and subwavelength imaging (*13*, *25*–*27*) are emerging.

However, the PhP response of a material is typically fixed once fabricated, owing to the inherent difficulty in modulating phonon modes. Despite its atomic thickness, graphene can efficiently modulate the v-HPhP response of films hundreds of nanometers thick, spanning out-of-plane modes in hBN (*28*) to in-plane modes in MoO_3_ (*29*–*33*) and twisted MoO_3_ (*34*), across terahertz to mid-infrared frequency range. This tunability originates from the hybridization between graphene plasmons and v-HPhPs. In particular, coupling with isotropic graphene plasmons drives a topological transition of the hyperbolic IFCs into elliptic ones as the graphene Fermi level increases (*29*–*31*, *34*). By engineering spatially varying doping profiles, electrically controlled negative refraction has been demonstrated at interfaces separating hyperbolic and elliptic IFC regions (*22*). More recently, experiments have demonstrated actively tunable v-HPhP nanoresonators (*35*, *36*) and their electrical readout (*36*), underscoring their potential for on-chip spectroscopic applications.

Nevertheless, the practical dynamic range of graphene’s doping level is restricted. Whereas chemical or charge transfer–induced doping strategies can yield relatively high but fixed Fermi levels (0.6 to 0.7 eV) (*30*, *37*, *38*), purely electrical gating in an hBN dielectric environment provides dynamic tunability with minimal additional plasmon damping (*36*, *39*–*41*), yet the Fermi level is typically limited to ~0.4 eV due to dielectric breakdown. Therefore, exploring the fundamental mechanism of v-HPhP tuning efficiency and approaching the ultimate limit are critical for advancing related applications. Current demonstrations of electrically tunable v-HPhPs rely critically on specific substrates such as SiO_2_ (*22*, *34*) and hBN (*28*). As will be shown here, this substrate dependence imposes a substantial constraint on further improving tuning efficiency. Here, the ultimate limit of tuning efficiency is revealed when graphene resides at the interface between the v-HPhP film and air. To realize this optimal configuration, we propose and experimentally demonstrate a general paradigm using interlayer-biased double-layer graphene (DLG). This approach not only maximizes the tuning efficiency but also extends the dynamic range by nearly twofold. As a representative, v-HPhPs of MoO_3_ and the canalized propagation are electrically tuned by varying the interlayer bias. Furthermore, by leveraging the enhanced flexibility of this electrical tuning, high-efficiency, angle-switchable directional in-plane electromagnetic energy transfer is demonstrated.

## RESULTS

### Tuning efficiency of v-HPhPs

**Fig. 1.**
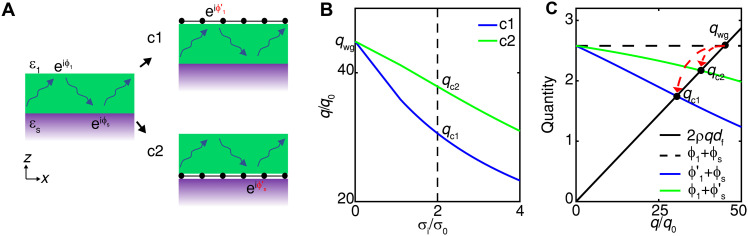
Comparison between tuning efficiency of HPhPs under different configurations. (**A**) Illustrations of the different cases. HPhP film is located within the two media of dielectric values ε_1_ and ε_s_. Waveguide modes propagate within the film, with the reflection phases of ϕ_1_ and ϕ_s_ at the top and bottom interfaces, respectively. The existence of graphene changes the reflection phases in two cases c1 and c2, where graphene exists at the interfaces of HPhP film-air and HPhP film-substrate, respectively. The reflection phases are modified as $\phi_{1}^{\prime }$ and $\phi_{s}^{\prime }$, respectively. (**B**) Dependence of the polariton momentum *q* at the [100] direction of MoO_3_ with optical conductivity of graphene σ_i_ for the two cases. $\sigma_{0}=e^{2}/4\hbar$. (**C**) *q*-dependent relations of the left term of Eq. 1, i.e., $2\rho qd_{f}$ (black solid), and the right terms with $\phi_{1}+\phi_{s}$ (black dashed), $\phi_{1}^{\prime }+\phi_{s}$ (blue solid) and $\phi_{1}+\phi_{s}^{\prime }$ (green solid) for the cases without graphene, c1, and c2, respectively. A value of σ_i_/σ_0_ = 2 is taken for the blue and green solid curves, corresponding to the black dashed line in (B). The light frequency is 931 cm^−1^, and the thickness of MoO_3_ is *d*_f_ = 100 nm. ε_1_ = 1 and ε_s_ = 5.

First, the general tuning efficiency of v-HPhPs by graphene plasmons is investigated theoretically, as shown in Fig.1. Generally, the v-HPhPs are represented by the opposite signs of real parts for the in-plane [represented by the *x* direction here, Re(ε*_x_*)] and out-of-plane [Re(ε*_z_*)] dielectric values, as studied in various materials such as hBN (*1*) and MoO_3_ (*8*, *9*). For the case without graphene, as illustrated in the left panel of Fig.1A, it is well known that the in-plane polariton momentum *q* for the HPhP waveguide modes satisfies (details in section S1)

$$2\rho qd_{f}=\phi_{1}+\phi_{2}+2m\pi ,m=0,1,2,\ldots$$ (1)

$$\phi_{1}=2\text{atan}\frac{\varepsilon_{1}}{\varepsilon_{z}\rho }$$ (2)

$$\phi_{s}=2\text{atan}\frac{\varepsilon_{s}}{\varepsilon_{z}\rho }$$ (3)

where *d*_f_ is the film thickness. The parameter ρ is described by $\rho =-i\sqrt{\frac{\varepsilon_{t}}{\varepsilon_{z}}}$, and ρ*q* gives the *z* component of the wave momentum. ϕ_1_ and ϕ_s_ are the reflection phases for the waveguide mode at the interfaces of film/air (ε_1_) and film/substrate (ε_s_), respectively. Here, only the first branch of the waveguide mode (*m* = 0) is considered, which dominates in experiment (*8*, *9*, *28*).

Two configurations where graphene lies at the interfaces of v-HPhP film/air (c1) and v-HPhP film/substrate (c2) are compared, with ε_1_ = 1, as illustrated by the right two panels in Fig. 1A. The latter case corresponds to the configurations in previous experiments (*22*, *34*), where the substrate of SiO_2_ or hBN is usually used and the Fermi level is tuned through back gate. Here, the general case where the substrate holds the dielectric value ε_s_ > 1 is studied. On the other hand, electrical tunability in the c1 case relies on the v-HPhP film acting as a suitable gate dielectric, which is usually not the case except for hBN (*28*, *39*). The existence of graphene plasmons modifies the reflection phase at the interface it lies within, from ϕ_1_ (ϕ_s_) to $\phi_{1}^{\prime }$ ($\phi_{s}^{\prime }$) for c1 (c2), with (details in section S1)

$$\phi_{1}^{\prime }=2\text{atan}\frac{\varepsilon_{1}-qS_{g}}{\varepsilon_{z}\rho }$$

$$\phi_{s}^{\prime }=2\text{atan}\frac{\varepsilon_{s}-qS_{g}}{\varepsilon_{z}\rho }$$

$$S_{g}=\frac{\sigma_{i}}{\varepsilon_{0}\omega }$$

where σ_i_ is the imaginary part of the optical conductivity of graphene. As a representative, Fig. 1B compares the dependence of the polariton momentum *q* in the [100] direction of MoO_3_ with σ_i_ between the two cases, with ε_s_ = 5. The light frequency is at ω = 931 cm^−1^, where v-HPhPs exist near the [100] direction of MoO_3_ (*8*, *9*). As σ_i_ increases, the polariton momentum decreases from $q_{\text{wg}}$ (without graphene) to $q_{c1}$ and $q_{c2}$ at σ_i_/σ_0_ = 2 for the two cases. The tuning efficiency, defined as the relative change in polariton momentum $\eta =∆q/q_{\text{wg}}$, reaches 32% in case c1 compared to 16% in case c2. Case c1 further maintains a consistently higher efficiency across all σ_i_. For a typical upper bound of $E_{F}$ = 0.4 eV (σ_i_/σ_0_ ≈ 4.4), the efficiency approaches approximately 60%. Even higher tuning efficiencies are achieved at elevated light frequencies and reduced film thicknesses (fig. S2).

The difference between tuning efficiencies of the two cases can be understood from the dispersion relations, which are plotted in Fig. 1C. The black solid curve plots the dependence of the left term ($2\rho qd_{f}$) in Eq. 1, which increases linearly with *q*. Besides, the right term in Eq. 1 ($\phi_{1}+\phi_{s}$) in the case without graphene is described by the horizontal line (black dashed). The intersection between the two curves gives the polariton momentum $q_{\text{wg}}$. On the other hand, the existence of graphene in the two cases introduces *q* dependence to the right term through $\phi_{1}^{\prime }$ for c1 and $\phi_{s}^{\prime }$ for c2, which are plotted as the blue and green solid curves for a value of σ_i_/σ_0_ = 2 (corresponding to the black dashed curve in Fig. 1B). Both curves decrease from the starting point at $\phi_{1}+\phi_{s}$. However, the slope is higher for the case c1 than c2, which generates a lower value of $q_{c1}$ than $q_{c2}$. The higher slope in c1 is intrinsically correlated with the smaller value of ε_1_ (being 1) than ε_s_ (being 5 here) appearing in $\phi_{1}^{\prime }$ and $\phi_{s}^{\prime }$, respectively, through the inverse tangent function (fig. S3). In practice, back gate (*22*, *34*) with dielectric value ε_s_ > 1 is usually necessary for tuning graphene electrically, which therefore obstructs the improvement of the tuning efficiency. Here, we demonstrate that by putting graphene at the interface between v-HPhP film and air, the highest tuning efficiency can be achieved. Nevertheless, achieving this tunability across diverse v-HPhP materials requires a fundamentally different electrical gating approach, which we investigate below.

### Principle of interlayer-biased DLG

**Fig. 2.**
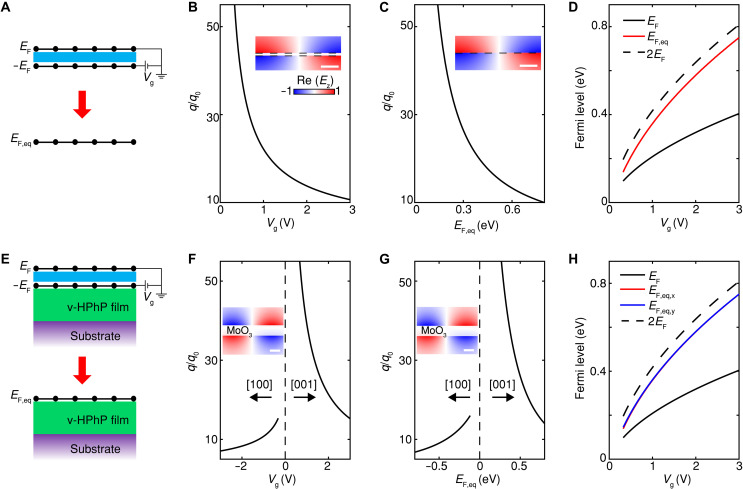
Principle for electrically tuned polariton dispersion by coupled DLG. (**A**) Proposed configuration consists of two graphene layers separated by a thin layer of dielectric (hBN here, with thickness of *d*). (**B**) Plasmon dispersion for the hybrid optical mode of the coupled DLG under $V_{g}$. Inset: Spatial distributions of the *z* component of the electrical field for the optical plasmon mode at $E_{F}$ = 0.1 eV. The two dashed lines label the DLG. (**C**) Plasmon dispersion for the individual graphene case with $E_{F,\text{eq}}$. Inset: Spatial distributions of the *z* component of the electrical field for the plasmon mode at $E_{F,\text{eq}}$ = 0.143 eV. The dashed line labels graphene. (**D**) Dependence of $E_{F}$ and $E_{F,\text{eq}}$ with $V_{g}$. A curve for 2$E_{F}$ is plotted as a reference. (**E** to **H**) Similar to (A) to (D), while for the cases where the coupled DLG lies above the v-HPhP film. Hybrid plasmon-polariton dispersions and the equivalent Fermi levels for the [100] and [001] directions of MoO_3_ are presented. The two curves for $E_{F,\text{eq},x}$ (red solid) and $E_{F,\text{eq},y}$ (blue solid) overlap with each other in (H). Insets in (F) and (G), spatial distributions of the *z* component of the electrical field for the polariton modes in the [100] direction of the DLG-MoO_3_ under $E_{F}$ = 0.128 eV (F) and graphene-MoO_3_ under $E_{F,\text{eq}}$ = 0.2 eV (G). The light frequency is 931 cm^−1^, the thickness of hBN is *d* = 3 nm, and MoO_3_ is 100-nm thick. The dielectric environment is air. Scale bars, 20 nm [(B) and (C)] and 100 nm [(F) and (G)].

We demonstrate then that the configuration c1 can be experimentally achieved through interlayer-biased DLG. The principle is illustrated in Fig. 2. As illustrated in Fig. 2A, the proposed configuration consists of two graphene layers separated by a thin layer of dielectric. Here, for the light frequencies away from phonon modes of hBN, hBN can serve as an excellent dielectric for tuning the Fermi levels $E_{F}$ and $-E_{F}$ under the interlayer bias $V_{g}$ applied between the two graphene layers (*41*, *42*). It is well known that plasmon hybridization in DLG results in optical and acoustic plasmon modes (*41*–*44*) (more discussions in section S2). Specifically, for the very thin interlayer space considered here, the acoustic mode carries a much higher polariton momentum than the optical mode (fig. S4). Consequently, the optical mode dominates the experimental observations (*41*, *42*, *44*). Based on this fact, we investigate the dispersion of the optical mode under varied $V_{g}$ with an interlayer space of *d* = 3 nm, as plotted in Fig. 2B. Here, by increasing $V_{g}$, the Fermi level $E_{F}$ is increased, which relationship is shown in Fig. 2D. Meanwhile, the plasmon momentum of the optical mode decreases with increasing $V_{g}$.

To elucidate the plasmonic behavior of the self-biased DLG, it can be equivalent to a hypothetical configuration consisting of one individual graphene layer with the tunable Fermi level $E_{F,\text{eq}}$, as illustrated in Fig. 2A. Plasmon momentum in the latter configuration is plotted in Fig. 2C, showing a monotonic decrease with $E_{F,\text{eq}}$. The equivalence between the two configurations is performed by matching the polariton momenta in Fig. 2, B and C. The equivalence is further established by the consistent spatial field distributions presented in the insets of Fig. 2, B and C. Except for the region between the DLG, periodic oscillations of *z* component of the electrical field with opposite signs on both sides of graphene are observed. The corresponding relation between $E_{F,\text{eq}}$ and $V_{g}$ is plotted in Fig. 2D. $E_{F,\text{eq}}$ increases with $V_{g}$ and consistently exceeds $E_{F}$, approaching nearly twice its value. The ratio $E_{F,\text{eq}}/E_{F}$ further grows with decreasing interlayer spacing and lower light frequency (fig. S4). In the zero-spacing limit and considering the optical plasmon mode, the coupled DLG effectively behaves as a single graphene sheet with an optical conductivity equal to the sum of those of the two graphene layers, yielding $E_{F,\text{eq}}\approx 2E_{F}$. However, for *d* approaching the atomic scale, the interlayer electron coupling effect will be notable and the electronic band structures would be modified. In this work, we consider only the interlayer plasmon coupling. *d* takes values higher than 1 nm to avoid the possible electron coupling effect.

Based on the equivalence with an individual graphene of tunable Fermi level, the self-biased DLG provides a general platform for tuning various polariton systems electrically. Specifically, it is used for realizing the configuration c1 shown in Fig. 1A, which exhibits the highest tuning efficiency of v-HPhPs. The equivalence principle is further elaborated in Fig. 2, E to H. By putting the coupled DLG on top of the v-HPhP film, the optical plasmon mode of graphene hybridizes with v-HPhPs, which results in tunable dispersion of the hybrid polaritons by the interlayer voltage $V_{g}$. As an example, Fig. 2F presents the dependence of the polariton modes in both the [100] and [001] directions of MoO_3_ with $V_{g}$. For the light frequency of 931 cm^−1^ here, v-HPhPs are supported near the [100] direction. Moreover, the positive dielectric value in the [001] direction introduces further in-plane hyperbolic properties (*8*, *9*), and distinct hybrid polariton dispersions are presented in the two directions. Similarly, polariton dispersions with $E_{F,\text{eq}}$ are plotted in Fig. 2G for the equivalent configuration (bottom of Fig. 2E), which is exactly c1 shown in Fig. 1. As expected, consistent spatial field distributions for the hybrid plasmon-HPhP modes in the [100] direction are observed in the insets of Fig. 2, F and G, where distributions of *z* component of the electrical field with opposite signs on both sides of MoO_3_ are presented. The corresponding dependence of $E_{F,\text{eq}}$ for the two directions is plotted in Fig. 2H. The values of $E_{F,\text{eq}}$ for the two directions are almost identical under varied $V_{g}$, and their dependence is consistent with that in the case without MoO_3_ in Fig. 2D. Therefore, the DLG functions as an equivalent self-biased graphene layer, enabling v-HPhP tunability even in anisotropic cases, without relying on specific dielectric environments. By putting it directly at the interface of v-HPhP film and air (configuration c1 in Fig. 1A), the highest tuning efficiency can be realized, with the added benefit of nearly doubled dynamic range.

### Experimental demonstration of interlayer-biased DLG

**Fig. 3.**
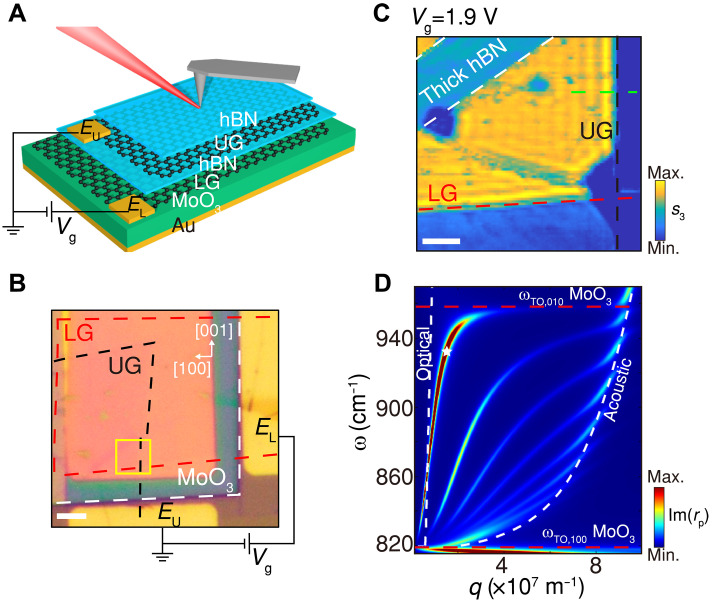
Experimental configuration. (**A**) Schematic of the device: hBN (2 nm)/graphene/hBN (2.3 nm)/graphene/MoO_3_ (126 nm)/Au. The UG layer is grounded through electrode $E_{U}$, while a gate voltage $V_{g}$ applies between the LG (via electrode $E_{L}$) and UG. An infrared laser at frequency of 931 cm^−1^ illuminates the metallic tip of s-SNOM, and near-field optical signals are measured during tip scans. (**B**) Optical micrograph of the fabricated sample. The UG (black dashed) and LG (red dashed) overlap above the MoO_3_ layer (white dashed) on the Au substrate. Both graphene layers are electrically connected to their respective electrodes as in (A). Scale bar, 3 μm. (**C**) Near-field amplitude *s*_3_ image of the yellow-marked region in (B), measured at $V_{g}$ = 1.9 V. The low-signal top-left area corresponds to thicker top hBN. Scale bar, 500 nm. (**D**) Polariton dispersion along the [100] direction of MoO_3_. White dashed lines indicate optical and acoustic polariton modes of the DLG stack (hBN/graphene/hBN/graphene). The two red dashed lines indicate the TO phonon along the [010] direction and the TO phonon along the [100] direction of MoO_3_, respectively. The white star marks the experimental data extracted from (C).

In the following, we demonstrate the above proposal in experiment. The device configuration is shown in Fig. 3, A and B. Using the dry-transfer process (Materials and Methods), the lower (LG) and upper (UG) graphene layers were sequentially placed onto the MoO_3_ layer supported by an Au substrate, with their boundaries outlined in Fig. 3B. The two layers are separated by an hBN spacer of about 2.3 nm thickness and electrically connected to Au/Pt electrodes $E_{U}$ and $E_{L}$, which enable the application of the interlayer voltage $V_{g}$. Modulating $V_{g}$ tunes the Fermi levels of the UG and LG within the overlapping region (formula in section S3.2). Notably, a very thin layer of hBN (around 2-nm thick) exists on the UG due to sample fabrication consideration. Its existence does not affect the principle of interlayer biasing and only decreases slightly the equivalent Fermi level as compared to the case without the top layer of hBN (fig. S4). Scattering-type scanning near-field optical microscopy (s-SNOM; Neaspec/Attocube) is used for measuring the polariton dispersions during tuning the interlayer bias in situ (Materials and Methods). Near-field amplitude signal (*s*_3_) is obtained by demodulation near the third harmonics of the tip oscillation frequency.

At zero bias, both graphene layers are nearly undoped, leading to opposite doping under an applied interlayer voltage (details in section S3.1). Figure 3C shows the measured near-field amplitude *s*_3_ image in the yellow-marked region of Fig. 3B at $V_{g}$ = 1.9 V. In the overlapping area between the UG and LG (outlined by the black and red dashed lines, respectively), their Fermi levels reach ±0.34 eV, while no electrical doping occurs outside this region. Consequently, polariton waves launched by the metallic tip are reflected at the graphene edges, and interference between incident and reflected polaritons produces the periodic fringes observed in Fig. 3C, with a period equal to half of the polariton wavelength (λ_p_). The mode near the UG edge corresponds to coupling between graphene plasmons and MoO_3_ v-HPhPs along the [100] axis, as shown in Fig. 3D. Here, the optical and acoustic polariton branches of the DLG stack (hBN/UG/hBN/LG, white dashed curves) hybridize with multiple waveguide modes of v-HPhPs within the TO phonon frequencies along the [100] and [010] directions (*8*, *9*), and the measured polariton wavelength (425 nm) agrees well with the first hybrid branch.

**Fig. 4.**
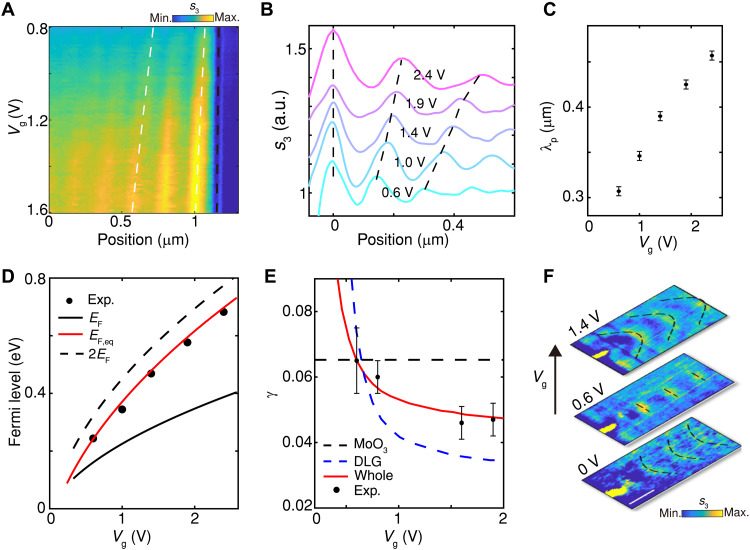
Interlayer voltage-tuned v-HPhPs. (**A**) Profiles of *s*_3_ across the UG edge (black dashed), taken along the green dashed line in Fig. 3C, for $V_{g}$ swept from 0.8 to 1.6 V. White dashed curves are guides to the eye. (**B**) Polariton profiles extracted at different $V_{g}$. (**C**) Dependence of the polariton wavelength λ_p_ on $V_{g}$. (**D**) Comparison between experimentally obtained $E_{F,\text{eq}}$ (black dots) and theoretical values of $E_{F}$, $E_{F,\text{eq}}$, and $2E_{F}$. (**E**) $V_{g}$-dependent polariton damping rates for MoO_3_ v-HPhPs (black dashed), the DLG stack (hBN/graphene/hBN/graphene, blue dashed), the whole heterostructure (red solid), and experimental data (dots). (**F**) Demonstration of $V_{g}$-tunable topological transitions, where the spatial wavefront of *s*_3_ excited by a Ag antenna (yellow strips in the left, see fig. S8) transits with $V_{g}$. The black dashed curves are guides to the eye. Scale bar, 500 nm. In (F), data are from a separate sample composed of hBN (2.9 nm)/graphene/hBN(4.2 nm)/graphene/MoO_3_(168 nm)/Au, measured at 897 cm^−1^. a.u., arbitrary unit.

Further modulation of $V_{g}$ tunes the polariton wavelength, as shown in Fig. 4 (A to C). The polariton fringe period systematically increases with $V_{g}$ from 0.8 to 1.6 V (Fig. 4A), consistent with the extracted polariton profiles at different voltages (Fig. 4B; corresponding *s*_3_ images in fig. S7). As summarized in Fig. 4C, the measured polariton wavelength grows from 310 nm at $V_{g}$ = 0.6 V to 457 nm at $V_{g}$ = 2.4 V, demonstrating efficient control of polariton momentum by interlayer bias.

The experimental results are directly compared with the equivalent model in Fig. 2. From the measured polariton wavelengths, the equivalent Fermi level is extracted (black dots, Fig. 4D), increasing from 0.23 to 0.68 eV as $V_{g}$ rises from 0.6 to 2.4 V. Theoretical calculations (red solid curve) based on the device geometry show excellent agreement with experiment, and both approach nearly twice the Fermi level of a single graphene layer (black dashed curve), validating the equivalent model in Fig. 2. The maximum equivalent Fermi level achieved here is about twice that of previous electrically gated graphene devices for tuning MoO_3_ v-HPhPs (*34*, *39*) and comparable to chemical or charge transfer–induced doping strategies (*30*, *37*, *38*) while retaining the essential advantage of electrical tunability. Moreover, the comparison of IFCs across multiple directions (fig. S8) confirms that this equivalence is independent of anisotropy as predicted in Fig. 2H.

The polariton damping rate, defined as γ=Im(q)/Re(q), is analyzed in Fig. 4E (detailed analysis in section S3.3). Together with the $V_{g}$-dependent Re(*q*) shown in Fig. 4C, these results reveal that the damping of the coupled system is also tuned by interlayer voltage. Specifically, the DLG stack (blue dashed) is expected to exhibit reduced damping with increasing $V_{g}$, away from the interband transition region and potentially even below that of MoO_3_ v-HPhPs (black dashed). Consequently, the damping rate of the coupled system (red solid) decreases with $V_{g}$, in good agreement with the experimentally extracted values (dots) and consistent with earlier reports on single-layer graphene tuning of v-HPhPs (*34*).

Compared with previous approaches that rely on specific dielectric substrates such as SiO_2_ (*22*, *34*), the self-biased configuration reported here is substrate-independent and simultaneously extends the tuning range by nearly twice. This capability enables the electrical control of topological transitions on Au substrates, which provides inherently flatter dispersion contours (*30*). The wavefront evolution in Fig. 4F directly illustrates this transition: from hyperbolic ($V_{g}$ = 0 V) to flat ($V_{g}$ = 0.6 V) and elliptic ($V_{g}$ = 1.4 V) patterns. Fourier analysis (fig. S8) further confirms the flattening of the IFC at $V_{g}$ = 0.6 V. Beyond this demonstration, the configuration is fully compatible with engineered dielectric platforms such as planar refraction (*19*) and directional polaritons in forbidden directions (*45*), offering a versatile route toward reconfigurable on-chip polaritonic devices.

### Angle-switchable directional energy propagation

**Fig. 5.**
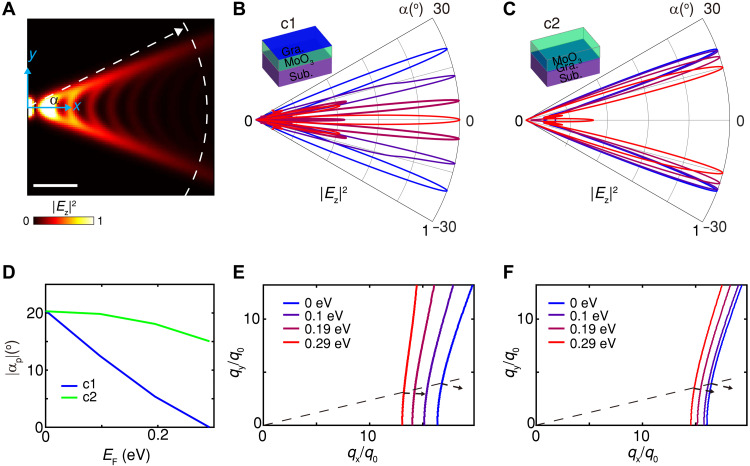
Switchable directional propagation. (**A**) Spatial distributions of ${|E_{z}|}^{2}$ above MoO_3_ under excitation by a vertically polarized point dipole of 100 nm above. The *x* and *y* axes correspond to the [100] and [001] directions of MoO_3_, respectively. The *x* and *y* coordinates of the dipole is (0,0). The directional distribution of ${|E_{z}|}^{2}$ at a distance of around 2 μm away from the source (marked by the white dashed curve) is extracted at angle α with respect to the *x* axis. Here, $E_{F}$ = 0 eV. Scale bar, 500 nm. (**B** and **C**) Extracted distribution of ${|E_{z}|}^{2}$ with α along the white dashed curve in (A) at varied values of $E_{F}$ for the cases c1 (B) and c2 (C). (**D**) Evolution of the absolute peak angle $|\alpha_{p}|$ in (B) and (C) with the Fermi level. (**E** and **F**) Corresponding IFCs for (B) and (C). The dashed arrows mark the energy flux directions at $E_{F}$ = 0 and 0.29 eV for a fixed momentum angle. The light frequency is 897 cm^−1^, and the thickness of MoO_3_ is 120 nm. ε_s_ takes a value of 5.

After demonstrating the principle of the coupled DLG and its flexibility in electrically tuning v-HPhPs, we present, in the following, its applications in tunable directional electromagnetic energy transfer at deep subwavelength. Recent studies have shown ray-like propagation of HPhPs (*11*, *46*, *47*). Yet, the dynamical control of in-plane energy direction at deep subwavelength in the mid-infrared range, a key requirement for applications such as on-chip thermal management and biosensing (*5*, *48*, *49*), has not been demonstrated previously. Figure 5A presents the spatial distribution of electrical field ${|E_{z}|}^{2}$ launched by a vertically polarized point dipole above MoO_3_, without graphene doping. Most of the optical energy propagates in two directions, which correspond to the energy flux directions near the asymptote of hyperbolic IFCs, consistent with previous studies (*8*, *9*). This behavior is further evidenced by the angle-dependent distribution extracted at around 2 μm from the source (white dashed curve in Fig. 5A), plotted in Fig. 5B, which shows a maximum near $|\alpha_{p}|\approx 20{}^{\circ}$ at $E_{F}$ = 0 eV.

The effect of graphene’s tunable Fermi level on the energy propagation direction is compared between the two configurations, c1 and c2 as described in Fig. 1. As shown in Fig. 5B for c1 where graphene lies at the interface of MoO_3_-air, the energy direction is switched from peak angle $|\alpha_{p}|\approx 20{}^{\circ}$ to $\alpha_{p}\approx 0{}^{\circ}$ with the increase of $E_{F}$ from 0 to 0.29 eV, as further plotted in Fig. 5D (blue curve). Experimentally, the far-field excitation of a metallic antenna can act as a polariton source, where excitation asymmetry (*46*, *50*) can be further exploited to break the mirror symmetry shown in Fig. 5A. A near-field detection scheme with suppressed tip-local response is essential for resolving directional propagation (see section S4.2 for details). In contrast, c2, with graphene at the MoO_3_-substrate interface, exhibits much weaker tunability over the same Fermi level range (Fig. 5C and green curve in Fig. 5D).

The dynamic tuning can be further understood from the IFC evolution (Fig. 5, E and F). As $E_{F}$ increases, the energy flux direction (black arrows) at a fixed momentum angle rotates progressively toward the horizontal due to coupling with graphene plasmons. Since c1 exhibits higher tuning efficiency as demonstrated in Fig. 1, the change in energy flux direction at a given Fermi level is more pronounced than in c2.

## DISCUSSION

In conclusion, we demonstrate that coupled DLG serves as a self-biased platform for flexible electrical tuning of v-HPhPs. This tunability is substrate-independent, with maximum efficiency achieved when graphene lies at the film-air interface, enabling distinct functionalities where dielectric engineering is critical (*19*, *30*, *45*). The enhanced tuning efficiency and extended dynamic range broaden applications ranging from resonance-frequency modulation (*36*) of v-HPhP nanostructures to strong light-matter interactions (*23*). Meanwhile, operating at voltages as low as 1 V, two orders of magnitude lower than prior work (*22*, *34*), this platform offers a practical route toward on-chip nanophotonics.

The strategy generalizes across HPhP systems from terahertz to mid-infrared, and its flexibility further enables tunable responses in multilayer v-HPhP heterostructures (*18*, *51*). Future demonstrations of negative refraction (*21*, *22*), beam steering (*19*, *20*, *52*, *53*), and subdiffractional imaging (*13*, *25*–*27*) with this large dynamic range could unlock transformative opportunities in nanophotonics. Moreover, electrically switchable directional energy transfer demonstrated here promises applications in directional thermal engineering (*49*) and on-chip biosensing (*5*).

## MATERIALS AND METHODS

### Sample fabrication

Layers of graphene, hBN, and α-MoO_3_ were obtained via mechanical exfoliation. Monolayer graphene was identified by the combination of optical reflection measurement and Raman spectrum. The heterostructure was assembled by the dry-transfer method using the polydimethylsiloxane (PDMS) stamp with polycarbonate (PC) film on top. First, the MoO_3_ layer was exfoliated on the PDMS stamp and transferred to the desired location of the substrate under guidance of optical microscopy. Subsequently, layers of hBN and graphene were picked up successively by the PDMS/PC stamp and released on top of MoO_3_, with one edge of graphene touching the prepatterned Au/Pd electrode on the substrate of SiO_2_/Si. The PC film was dissolved in chloroform. Last, another stack of hBN/graphene was caught up and released on top, with one edge of this layer of graphene lying on another electrode. An 0.01 M cetyltrimethylammonium chloride solution containing Ag nanowires (Advanced Plasmonic Technologies) was diluted 1:15 with deionized water, drop-casted onto a PC film, and allowed to dry naturally under ambient conditions. The PC film was then mounted onto a PDMS stamp and subsequently used to transfer the nanowires to the desired locations on the heterostructures, following a procedure similar to that used for two-dimensional material transfer.

### Near-field optical measurement

s-SNOM (Neaspec/Attocube) was used for measuring the near-field response with the interlayer bias applied in situ. A ^13^CO_2_ laser acted as the light source. Under the laser illumination, evanescent optical fields from the metallic tip (radius of around 20 nm) launched polariton fields on the heterostructures. The near-field optical signals were scattered to the far field by the metallic tip. The metallic tip oscillated at frequency of around 250 kHz, and the near-field signals were demodulated by the pseudo-heterodyne approach (*54*), where the reference arm was modulated simultaneously. In situ bias was applied through a voltage source (Keithley 2400), and the electrodes on the sample were wire-bonded to a printed circuit board.

## Supplementary Materials

### This PDF file includes:

Sections S1 to S4 Figs. S1 to S9 Table S1 References
