# Supplementary material for: Ultimate tuning of hyperbolic phonon polaritons
Source: Sci Adv. 2025 Dec 12;11(50):eadz6278. doi: 10.1126/sciadv.adz6278 (PMC12700210; doi:10.1126/sciadv.adz6278)
Supplement: Supplementary file 1 — Sections S1 to S4 Figs. S1 to S9 Table S1 References [file sciadv.adz6278_sm.pdf]

Science Advances
AAAS

Supplementary Materials for

Ultimate tuning of hyperbolic phonon polaritons

Linglong Zhang *et al.*

Corresponding author: Weiwei Luo, weiwei.luo@nankai.edu.cn; Wei Cai, weicai@nankai.edu.cn; Jingjun Xu, jjxu@nankai.edu.cn

Sci. Adv. **11**, eadz6278 (2025)
DOI: 10.1126/sciadv.adz6278

This PDF file includes:

Sections S1 to S4
Figs. S1 to S9
Table S1
References

1 Polariton dispersion calculations

1.1 Transfer matrix method (TMM)

In isotropic media, the electric field of the TM polariton mode with in-plane momentum q is in the form of

$$\mathbf{E} = \frac{E_0}{q}\begin{pmatrix} q_x \\ q_y \\ \pm iq^2/q_z \end{pmatrix} e^{i(q_x x + q_y y)} e^{\mp q_z^T z}, \tag{S1}$$

where

$$q_z = q_z^T = \sqrt{q^2 - \varepsilon q_0^2} \tag{S2}$$

and q_x and q_y are the x and y component of the in-plane momentum, respectively.

For the biaxial crystals like MoO_3, under the high-wavevector approximation $q/q_0 \gg 1$, the corresponding electric field can be expressed in the same way (*55*), with

$$q_z^T = \sqrt{\frac{\varepsilon_t}{\varepsilon_z}}q, \tag{S3}$$

$$q_z = \frac{q^2}{q_z^T}, \tag{S4}$$

$$\varepsilon_t = \varepsilon_x \cos^2\theta + \varepsilon_y \sin^2\theta \tag{S5}$$

and θ is the angle between the in-plane momentum and the [100] direction of MoO_3 (in x axis).

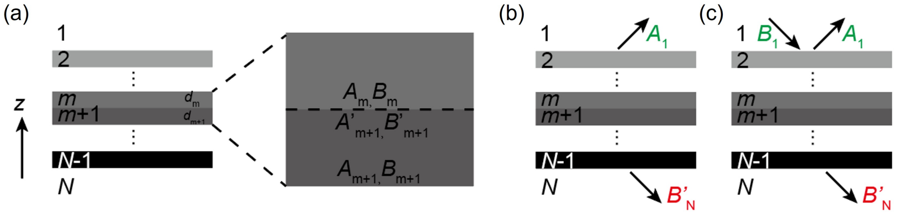

Figure S1: Illustrations for TMM. (a) Media are labeled as $j = 1, 2, ..., N-1, N$ from top to bottom in the heteostructure. The components A_m,B_m,A'_{m+1},B'_{m+1} exist at the interface of media m and $m+1$. (b,c) Schemes for calculating the eigen-mode of electric field distributions (b) and reflection coefficient r_p (c).

Accordingly, for the heterostructure with media labeled as $j = 1, 2, ..., N-1, N$ from top to

bottom (as illustrated in Fig.S1a), the electric field within media j is described as

$$\mathbf{E_j} = a_j \begin{pmatrix} q_x \\ q_y \\ +iq^2/q_{z,j} \end{pmatrix} \mathrm{e}^{i(q_x x + q_y y)} \mathrm{e}^{-q^T_{z,j} z} + b_j \begin{pmatrix} q_x \\ q_y \\ -iq^2/q_{z,j} \end{pmatrix} \mathrm{e}^{i(q_x x + q_y y)} \mathrm{e}^{+q^T_{z,j} z}. \tag{S6}$$

We can define $A_m = a_m \mathrm{e}^{-q^T_{z,j} z_{m,m+1}}$, $B_m = b_m \mathrm{e}^{+q^T_{z,j} z_{m,m+1}}$, $A'_{m+1} = a_{m+1} \mathrm{e}^{-q^T_{z,j} z_{m,m+1}}$ and $B'_{m+1} = b_{m+1} \mathrm{e}^{+q^T_{z,j} z_{m,m+1}}$ where $z_{m,m+1}$ is the value of z at the interface between the m and $m+1$ media ($m = 1, 2, ..., N-1$). According to the continua of the in-plane electric field and out-plane displacement field,

$$A_m + B_m = A'_{m+1} + B'_{m+1},$$
$$\frac{\varepsilon_{z,m}}{q_{z,m}}(A_m - B_m) = \frac{\varepsilon_{z,m+1}}{q_{z,m+1}}(A'_{m+1} - B'_{m+1}).$$

It can be obtained that

$$\begin{bmatrix} A_m \\ B_m \end{bmatrix} = \frac{1}{2} \begin{bmatrix} 1 + x_{m,m+1} & 1 - x_{m,m+1} \\ 1 - x_{m,m+1} & 1 + x_{m,m+1} \end{bmatrix} \begin{bmatrix} A'_{m+1} \\ B'_{m+1} \end{bmatrix} = M_{m,m+1} \begin{bmatrix} A'_{m+1} \\ B'_{m+1} \end{bmatrix}$$

where $x_{m,m+1} = \frac{\varepsilon_{z,m+1} q_{z,m}}{\varepsilon_{z,m} q_{z,m+1}}$. Meanwhile,

$$\begin{bmatrix} A'_{m+1} \\ B'_{m+1} \end{bmatrix} = \begin{bmatrix} \tau_{m+1} & 0 \\ 0 & \tau^{-1}_{m+1} \end{bmatrix} \begin{bmatrix} A_{m+1} \\ B_{m+1} \end{bmatrix} = T_{m+1} \begin{bmatrix} A_{m+1} \\ B_{m+1} \end{bmatrix} \tag{S7}$$

where $\tau_{m+1} = \mathrm{e}^{-q^T_{z,m+1} d_{m+1}}$. Here, d_{m+1} is the thickness of media $m+1$ (applicable for $m = 1, 2, ..., N-2$).

Generally, the parameters at the top media ($j = 1$) and the bottom media ($j = N$) are connected through

$$\begin{bmatrix} A_1 \\ B_1 \end{bmatrix} = S \begin{bmatrix} A'_N \\ B'_N \end{bmatrix} = \begin{bmatrix} Q(1,1) & Q(1,2) \\ Q(2,1) & Q(2,2) \end{bmatrix} \begin{bmatrix} A'_N \\ B'_N \end{bmatrix}$$

where $Q = M_{1,2} T_2 M_{2,3} T_3 ... T_{N-1} M_{N-1,N}$ is a 2×2 matrix.

For calculating the eigen-mode of the electric field distribution (Fig.S1b), it has $B_1 = 0$ and $A'_N = 0$. So the polariton dispersion can be solved from

$$Q(2,2) = 0. \tag{S8}$$

On the other hand, the reflection coefficient $r_p = A_1/B_1$ can be calculated under the condition that $A'_N = 0$.

The dielectric response of α-MoO_3 is described using the Lorentz model

$$\varepsilon_j = \varepsilon_j^{\infty}(1 + \frac{\omega_{LO,j}^2 - \omega_{TO,j}^2}{\omega_{TO,j}^2 - \omega^2 - i\omega\Gamma_j}), j = x, y, z, \tag{S9}$$

where ε^{∞} is the high frequency dielectric constant, ω_{TO} and ω_{LO} are the transverse (TO) and longitude (LO) optical phonon frequencies, respectively, and Γ is the broadening factor. Parameter values are adopted from previous studies (*9*), as shown in Tab.S1.

j	ε_j^{∞}	$\omega_{LO,j}(cm^{-1})$	$\omega_{TO,j}(cm^{-1})$	$\Gamma_j(cm^{-1})$
x ([100])	4.0	972	820	4
y ([001])	5.2	851	545	4
z (010)	2.4	1004	958	2

Table S1: Dielectric parameters of MoO_3 in the mid-infrared range.

1.2 Treatment of graphene in TMM

In Maxwell's equations,

$$\nabla \times \mathbf{H} = \mathbf{J} + \frac{\partial}{\partial t}\mathbf{D}.$$

For polariton modes with wavevector in x direction, $\mathbf{B} = B_y \mathrm{e}^{iqx}\mathrm{e}^{-q_z|z|}\hat{\mathbf{y}}\mathrm{e}^{-i\omega t}$. The relationship between B_y and D_z reads

$$B_y = -\frac{\omega\mu_0}{q}D_z.$$

If graphene with optical conductivity σ lies at the interface of media $j = m$ and $j = m + 1$, the boundary condition reads

$$B_{m,y} - B_{m+1,y} = -\mu_0\sigma J_x = -\mu_0\sigma E_x.$$

Therefore,

$$D_{m,z} = D_{m+1,z} + \frac{\sigma}{\varepsilon_0\omega}qE_x.$$

The boundary conditions are expressed as

$$A_m + B_m = A'_{m+1} + B'_{m+1},$$
$$\frac{\varepsilon_{z,m}}{q_{z,m}}(A_m - B_m) = \frac{\varepsilon_{z,m+1}}{q_{z,m+1}}(A'_{m+1} - B'_{m+1}) - \frac{i\sigma}{\varepsilon_0\omega}(A_m + B_m).$$

Accordingly,

$$\begin{bmatrix} A_m \\ B_m \end{bmatrix} = \frac{1}{2}\begin{bmatrix} 1 + x_{m,m+1} + P_m & 1 - x_{m,m+1} + P_m \\ 1 - x_{m,m+1} - P_m & 1 + x_{m,m+1} - P_m \end{bmatrix}\begin{bmatrix} A'_{m+1} \\ B_{m+1'} \end{bmatrix}$$

where

$$P_m = \frac{q_{z,m}}{\varepsilon_{z,m}}S_g,$$
$$S_g = \frac{-i\sigma}{\varepsilon_0\omega}.$$

The optical conductivity σ of graphene is described by the Kubo formula (*56*), including both intraband and interband contributions,

$$\begin{aligned} \sigma &= \sigma_{intra} + \sigma_{inter} \\ \sigma_{intra} &= \frac{2e^2k_BT}{\pi\hbar^2}\frac{i}{\omega + i\tau^{-1}}\ln[2\cosh\frac{E_F}{2k_BT}] \\ \sigma_{inter} &= \frac{ie^2(\omega + i\tau^{-1})}{\pi\hbar}\int_0^{\infty}\frac{f(-\xi) - f(\xi)}{(\omega + i\tau^{-1})^2 - 4\xi^2}d\xi \end{aligned} \quad \text{(S10)}$$

Here, $f(\xi) = \frac{1}{e^{(\hbar\xi - E_F)/(K_BT)} + 1}$ is the Fermi-Dirac distribution. τ is the scattering time, and room temperature T=300 K is taken. This formula is used to calculate the polariton damping rate. We take $\tau = 0.2$ ps, corresponding to a graphene plasmon damping rate $\gamma = \omega\tau \approx 0.03$ at high doping, consistent with previous studies of hBN-encapsulated graphene at room temperature (*39*) .

For graphene Fermi levels exceeding the phonon energy, the optical conductivity is well described by the Drude formula as $\sigma_i = \sigma_0\frac{4}{\pi}\frac{E_F}{\hbar\omega}$ with $\sigma_0 = e^2/4\hbar$. When calculating polariton dispersion including the acoustic mode in coupled DLG, nonlocal effects must be considered (*57*). This is accounted for by introducing a correction factor $f(q/q_F)$ in the Drude formula:

$$f(z) = \frac{2}{z^2}[(1 - z^2)^{-1/2} - 1]$$

1.3 Polariton dispersion formula for the air/graphene/MoO_3/substrate heterostructure

For the case of air/graphene/MoO_3/substrate, since only the dielectric value of MoO_3 is anisotropic, we use ε_z as the dielectric value of MoO_3 in z direction, ε_1=1 for the air, ε_s for the substrate and $d_2 = d_f$ is the thickness of the MoO_3 layer. Under the quasi-static approximation, it has $q_{z,1} = \sqrt{q^2 - \varepsilon_1 q_0^2} \approx q$ and $q_{z,3} = \sqrt{q^2 - \varepsilon_s q_0^2} \approx q$. Here, the matrix Q is expressed as

$$Q = M_{1,2}T_2M_{2,3} = \frac{1}{4}\begin{bmatrix} 1 + x_{1,2} + P_1 & 1 - x_{1,2} + P_1 \\ 1 - x_{1,2} - P_1 & 1 + x_{1,2} - P_1 \end{bmatrix}\begin{bmatrix} \tau_2 & 0 \\ 0 & \tau_2^{-1} \end{bmatrix}\begin{bmatrix} 1 + x_{2,3} & 1 - x_{2,3} \\ 1 - x_{2,3} & 1 + x_{2,3} \end{bmatrix}$$

The polariton dispersion is calculated from

$$Q(2,2) = \tau_2(1 - x_{1,2} - P_1)(1 - x_{2,3}) + \tau_2^{-1}(1 + x_{1,2} - P_1)(1 + x_{2,3}) = 0, \tag{S11}$$

where

$$\begin{aligned} x_{1,2} &= \frac{\varepsilon_z q_{z,1}}{\varepsilon_1 q_{z,2}} = \frac{\varepsilon_z}{\varepsilon_1}\sqrt{\frac{\varepsilon_t}{\varepsilon_z}}, \\ x_{2,3} &= \frac{\varepsilon_s q_{z,2}}{\varepsilon_z q_{z,3}} = \frac{\varepsilon_s}{\varepsilon_z\sqrt{\frac{\varepsilon_t}{\varepsilon_z}}}, \\ \tau_2 &= e^{-q_{z,2}^T d_f} = e^{-\sqrt{\frac{\varepsilon_t}{\varepsilon_z}} q d_f}, \\ P_1 &= \frac{q_{z,1}}{\varepsilon_1} S_g = \frac{q}{\varepsilon_1} S_g. \end{aligned}$$

Eq.S11 can be expressed as

$$\begin{aligned} \tau_2^2 = -\frac{(1 + x_{1,2} - P_1)(1 + x_{2,3})}{(1 - x_{1,2} - P_1)(1 - x_{2,3})} &= \frac{1 + x_{2,v}}{1 - x_{2,v}} \cdot \frac{1 + x_{2,3}}{1 - x_{2,3}} \\ x_{2,v} &= \frac{1 - P_1}{x_{1,2}}. \end{aligned} \tag{S12}$$

Mathematically, one has

$$\frac{1 + x}{1 - x} = \mathrm{e}^{i2\phi},$$

where

$$\phi = \mathrm{atan}\frac{x}{i}.$$

Therefore, Eq.S12 would be

$$e^{-2\sqrt{\frac{\varepsilon_t}{\varepsilon_z}}qd_f} = e^{i2(\phi_a+\phi_b)}, \tag{S13}$$

$$\phi_a = \text{atan}\frac{1-P_1}{x_{1,2}i} = \text{atan}\frac{\varepsilon_1 - S_g q}{i\varepsilon_z\sqrt{\frac{\varepsilon_t}{\varepsilon_z}}}, \tag{S14}$$

$$\phi_b = \text{atan}\frac{x_{2,3}}{i} = \text{atan}\frac{\varepsilon_s}{i\varepsilon_z\sqrt{\frac{\varepsilon_t}{\varepsilon_z}}}. \tag{S15}$$

By defining $\rho = -i\sqrt{\frac{\varepsilon_t}{\varepsilon_z}}$, the polariton dispersions can be obtained from

$$\rho q d_f = \text{atan}\frac{\varepsilon_1 - S_g q}{\varepsilon_z\rho} + \text{atan}\frac{\varepsilon_s}{\varepsilon_z\rho} + l\pi, l = 0, 1, 2, .. \tag{S16}$$

This expression can be understood from the waveguide theory. In the case that Re($\varepsilon_t\varepsilon_z$) <0, which is considered in the main text, the polariton modes of MoO_3 would be the waveguide mode, with the z-component wave momentum as $k_z = \sqrt{-\frac{\varepsilon_t}{\varepsilon_z}}q = \rho q$. According to the waveguide theory, the eigen-modes satisfy

$$2k_z d_f = \phi_1' + \phi_s + 2m\pi, m = 0, 1, 2, ... \tag{S17}$$

where ϕ_1' and ϕ_s are the reflection phases at the interfaces of MoO_3-air and MoO_3-substrate, respectively. By comparing Eqs.S17 and S16, one gets

$$\phi_1' = 2\text{atan}\frac{\varepsilon_1 - S_g q}{\varepsilon_z\rho}, \tag{S18}$$

$$\phi_s = 2\text{atan}\frac{\varepsilon_s}{\varepsilon_z\rho}. \tag{S19}$$

Actually, the expressions for ϕ_1' and ϕ_s can be also obtained from calculating the reflection coefficients at the two interfaces. Similarly, the expressions of ϕ_1 and ϕ_s' can be obtained by switching between ε_1 and ε_s for the case when graphene lies at the interface of MoO_3-substrate. Besides, for the case of substrate of Au, one has $\phi_s = \pi/2$.

2 Further analysis of the equivalent model

Fig.S4a shows the hybrid plasmons in coupled double-layer graphene (DLG). The hybridization between plasmons in each individual graphene (black dashed curve in Fig.S4a) results in mode splitting into the acoustic (higher momentum) and optical (lower momentum) branches. The splitting

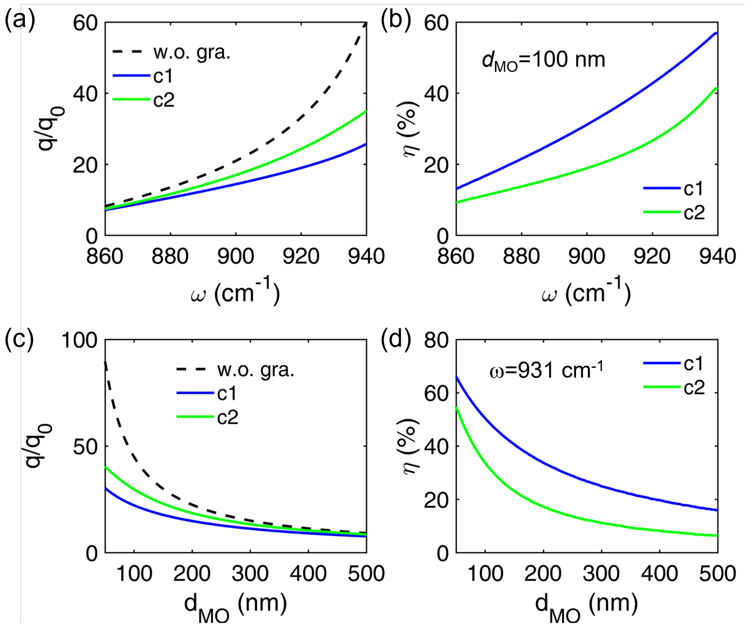

Figure S2: Dependence of tuning efficiency on light frequency (a,b) and film thickness (c,d). (a) Calculated polariton momentum for the cases without graphene (black dashed), c1 (blue) and c2 (green). (b) Corresponding tuning efficiency η as a function of light frequency for the two cases. The film thickness is fixed at 100 nm. (c,d) Same as (a,b), but for varied film thickness, with a fixed light frequency of 931 cm^{-1}. The Fermi level is set to E_F=0.4 eV.

gets stronger with the decrease of interlayer space d. Spatial distributions of Re(E_z) for these two modes are presented in Fig.S4b. For the acoustic mode, E_z is mostly confined within the two graphene layers, being in-phase outside of the two graphene layers. On the other hand, E_z distributes mostly outside of the two graphene layers, oscillating out of phase for the optical mode. For an interlayer space of 3 nm, the acoustic mode exhibits significantly higher polariton momentum than the optical mode (Fig.S4c).

Moreover, stronger interlayer coupling at smaller spacing leads to an increase of the ratio $E_{F,eq}/E_F$ with decreasing d (Fig.S4d). At lower light frequencies, the graphene plasmon wavelength becomes larger, enhancing interlayer coupling and thus further increasing $E_{F,eq}/E_F$ for a fixed d, as illustrated at 380 cm^{-1} (within the v-HPhP range of α-MoO_3 in the terahertz regime (*58*)).

Fig.S4e compares the damping rate of the optical mode with that of plasmons in individual graphene layer. Nearly identical rates are observed for an air spacer, consistent with the equivalence

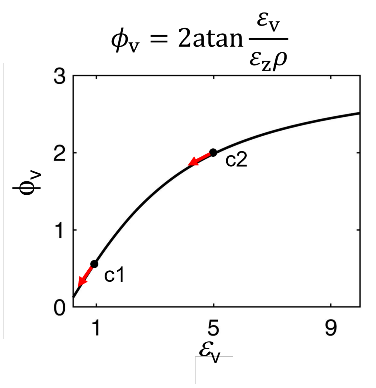

Figure S3: Dependence of the inverse tangent function $\phi_v = 2\mathrm{atan}\frac{\varepsilon_v}{\varepsilon_z\rho}$ on the dielectric parameter ε_v, where slopes of the cases c1 (ε_v=1) and c2 (ε_v=5) are labeled. The light frequency is 931 cm^{-1}.

between coupled DLG and a single layer. For a practical hBN spacer (blue curve), the optical mode damping increases slightly due to the dielectric loss of hBN.

Fig.S4f shows that the top hBN layer (2 nm) slightly reduces the equivalent Fermi level by less than 0.05 eV in the experimental sample of Fig. 3.

3 Extra experimental results and analysis

3.1 Determination of initial graphene doping

Determining the initial doping of graphene at zero bias is crucial for establishing the Fermi level dependence on gate voltage. From the near-field optical measurements in Fig. S5, we confirm that both graphene layers are initially nearly undoped.

Fig.S5a shows near-field images of the DLG–MoO_3 heterostructure across the green dashed line in Fig.3c of the main text. As V_g is swept from 1.6 to -1.6 V, the polariton fringe periods decrease and increase symmetrically around $V_g = 0$. This symmetry is further illustrated in Fig.S5b, where consistent polariton fringes are observed for opposite values of V_g at $|V_g| = 0.9$ and 1.5 V. Meanwhile, a local maximum in the near-field amplitude signal within the DLG region at $V_g \approx 0$ is observed, consistent with the expected near-field optical response of graphene at zero doping, arising from maximized interband transitions (*41*). Notably, polariton fringes are absent for $|V_g| < 0.5$ V due to reduced reflection at the UG edge when polariton wavelengths on both sides of the edge are

similar. This effect is not due to increased damping, as polariton features are still observable for heavily damped cases (*59, 60*).

Negligible initial doping is further confirmed for each graphene layer individually (Fig.S5c–f). Consistent fringes are observed in regions with and without the LG layer in the absence of the UG (Fig.S5c,d), indicating negligible LG doping. This is consistent with previous studies showing minimal doping of graphene by α-MoO_3 (*29*). Polariton damping is slightly higher in the LG region, as expected for zero-doping graphene/MoO_3 (*34*). Similar results are observed for the UG (Fig. S5e,f), as encapsulation by hBN isolates it from environmental doping (*39*). Owing to negligible doping from α-MoO_3 and the common use of hBN as a dielectric for graphene biasing, zero initial doping—and hence symmetric Fermi levels between the two layers—is readily achievable.

3.2 Dependence of the Fermi level on interlayer bias

For the very thin interlayer space (around 2 nm) in this work, the quantum capacitance effect (*57, 61*) is significant, which is considered in calculating the dependence of E_F and V_g. The relationship is expressed as

$$V_g = 2E_F + \frac{en_{\mathrm{dop}}}{C} \tag{S20}$$

$$E_F = \hbar v_F \sqrt{\pi n_{\mathrm{dop}}} \tag{S21}$$

$$\frac{1}{C} = \frac{d_{BN}}{\varepsilon\varepsilon_0} + \frac{d_{vac}}{\varepsilon_0}. \tag{S22}$$

Here, the first and second right terms of the first equation correspond to the quantum and geometry capacitance contributions, respectively, where n_{dop} is the doped carrier density of graphene. In the expression of the geometry capacitance C, the first right term represents the contribution from the interlayer hBN with the thickness of d_{BN} and dielectric value of ε=3.56. The second term is related to vacuum contribution, which is also considered in previous experimental study of nonlocal graphene plasmons under very thin interlayer space (*57*). It acts as a best guess as to the true correction required for an accurate atomic-scale capacitance, with the thickness of d_{vac}=0.17 nm.

3.3 Polariton damping analysis

Polariton damping rates are calculated by numerically solving the dispersion equation (Eq.S8) with complex polariton momenta. Fig.S6a shows the optical conductivity σ of graphene with E_F from Eq.S10. The real part of σ is dominated by interband transitions, significant for $E_{F,0} < \hbar\omega$ (around 0.1 eV in this work, indicated by the yellow dashed line). Accordingly, graphene plasmon damping decreases sharply for $E_F > E_{F,0}$ and further diminishes with increasing E_F (blue dashed curve in Fig. S6b), reaching ≈0.03 at high doping for a scattering time of $\tau = 0.2$ ps in Eq.S10, consistent with studies of hBN-encapsulated graphene (*39*), and lower than that of MoO_3 v-HPhPs.

Hybridization between graphene plasmons and v-HPhPs reduces polariton damping with increasing E_F, in agreement with recent experimental observations in graphene–MoO_3 heterostructures (*34*). As a result, the damping rate of the coupled heterostructure can be lower than that of v-HPhPs alone. Moreover, case c1 (described in Fig.1 of the main text) exhibits lower damping at high Fermi levels compared to case c2, consistent with its higher tuning efficiency.

Damping rates for the experimental configuration are also calculated using the E_F–V_g relation (Fig. S6c), with results shown in Fig.4e of the main text and Fig.S6d. Experimental near-field amplitude s_3 profiles are fitted using (*8, 39, 40*)

$$s_3(x) = \left|\frac{Ae^{i2qx(1+i\gamma)}}{\sqrt{x}} + B\right| \tag{S23}$$

where the first term represents polariton waves reflected from a sharp boundary at $x = 0$, and the second term accounts for local near-field response detected by the s-SNOM tip away from any reflector or other source. Here, experimental profiles across the sharp UG edge are used to ensure fitting accuracy. Notably, local defects and contaminants existing on the near-field response add uncertainties in the extracted damping rates. For the calculation, higher damping values of $\Gamma_x = \Gamma_z =$ 4.5 cm^{-1} in the Lorentz model of MoO_3 are adopted, yielding a damping rate of $\gamma_0 \approx 0.065$ (black dashed line in Fig.S6d), consistent with the experimentally extracted value of 0.062 near the MoO_3 edge without graphene.

3.4 Additional experimental results

Fig.S7a shows the same optical micrograph as in Fig.3b of the main text. Near-field optical images were acquired in two regions, labeled A and B, where region B corresponds to the area measured in

Fig.3c of the main text, and region A is located near the UG edge. Fig.S7b–f present the measured s_3 images at different interlayer voltages (V_g=0.6 to 1.9 V for region A, and V_g= 2.4 V for region B). During sample assembly, bubbles and wrinkles formed on the heterostructure, which appear in the near-field images and complicate the extraction of polariton profiles. To mitigate this, profiles were extracted along locations free of obvious defects. Notably, the influence of such defects can be greatly reduced using cleaning techniques like AFM brooming (*62*). Conversely, straight and sharp wrinkles can serve as effective reflection boundaries for extracting polariton profiles, as shown in Fig.S7. The polariton profiles presented in Fig.4b of the main text were extracted near both the black dashed lines corresponding to wrinkles (Fig.S7b–e) and the UG edge (Fig.S7f).

Fig. S8a and b presents additional experimental results for measuring the IFCs at V_g= 1.6 V. The near-field s_3 image (a) was acquired in region A indicated in Fig.S7a. Reflected polariton profiles were extracted along the dashed lines in different directions, and the resulting IFC agrees well with theoretical calculations (b).

Fig.S8c shows the AFM image of the sample investigated in Fig.4f of the main text. An Ag antenna (3 μm length×150 nm width ×80 nm height) is placed on the heterostructure to launch polariton waves under far-field illumination. Fig.S8d–f display the corresponding raw spatial near-field images (left panels) measured from the red dashed region in (c) at different V_g. The Fourier-transformed IFCs (right panels) reveal a transition from hyperbolic (0 V) to flat (0.6 V) and elliptic (1.4 V) contours, which are in good agreement with theoretical predictions (black solid curves).

4 More details on directional energy propagation

4.1 Analytical calculations of electrical field distributions excited by a vertically polarized point dipole

Three-dimensional simulations of electromagnetic field distributions of graphene-MoO_3 can be quite time-consuming. Instead, the analytical calculation method from the Dyadic Green's functions (*63*) is employed in the calculations of Fig.4 of the main text.

The top surface of the graphene-MoO_3 heterostructure is defined as z=0. The z-polarized point dipole with moment of (0,0,β) locates at $\mathbf{r} = (0, 0, z_0)$. E_z distributions at $\mathbf{R} = (x, y, z)$ above MoO_3

($z > 0$) is expressed as $E_z = E_{z,0} + E_{z,r}$, where $E_{z,0}$ is the one emitted directly by the dipole and $E_{z,r}$ is the one reflected by the heterostructure. According to the Dyadic Green's function theory, one has

$$E_{z,0} = \omega \mu_0 \beta G_0 \tag{S24}$$

$$G_0 = \frac{i}{8\pi^2 q_1^2} \iint_{-\infty}^{+\infty} \frac{q_x^2 + q_y^2}{q_{z,1}} e^{i[q_x x + q_y y + q_{z_1} |z - z_0|]} dq_x dq_y \tag{S25}$$

where $q_1 = \varepsilon_1 q_0$ is the wave momentum in the media above MoO_3. q_x, q_y and $q_{z,1}$ are the different components, satisfying $q_x^2 + q_y^2 + q_{z,1}^2 = q_1^2$. On the other hand, reflection from the polariton heterostructure is dominated by the p-component reflection coefficient r_p, which gives

$$E_{z,r} \approx \omega \mu_0 \beta G_p \tag{S26}$$

$$G_p = \frac{i}{8\pi^2 q_1^2} \iint_{-\infty}^{+\infty} r_p \frac{q_x^2 + q_y^2}{q_{z,1}} e^{i[q_x x + q_y y + q_{z_1} (z + z_0)]} dq_x dq_y \tag{S27}$$

Accordingly, the spatial distributions of E_z above MoO_3 can be calculated from

$$E_z \approx \omega \mu_0 \beta G \tag{S28}$$

$$G = G_0 + G_p = \frac{i}{8\pi^2 q_1^2} \iint_{-\infty}^{+\infty} \frac{q_x^2 + q_y^2}{q_{z,1}} e^{i(q_x x + q_y y)} [r_p e^{i q_{z_1} (z + z_0)} + e^{i q_{z_1} |z - z_0|}] dq_x dq_y. \tag{S29}$$

In polar coordinates, $\mathbf{R} = (r, \alpha, z)$ (r and α are the radius and angle, respectively) and the in-plane momentum $\mathbf{q} = (q_x, q_y)$ is replaced by $\mathbf{q} = (q, \theta)$. One has

$$G = \frac{i}{8\pi^2 q_1^2} \int_{-\infty}^{+\infty} dq \int_0^{2\pi} d\theta \frac{q^3}{q_{z,1}} e^{i[qr(\cos(\theta - \alpha))]} [r_p e^{i q_{z_1} (z + z_0)} + e^{i q_{z_1} |z - z_0|}] \tag{S30}$$

4.2 Discussion on experimental detection scheme

The observation of switchable directional propagation remains a major challenge in s-SNOM, as the strong local near-field response of the AFM tip dominates the detected signal and obscures the directional energy transport predicted in simulations.

As shown in Fig.S9a, a typical near-field amplitude s_3 image of hyperbolic phonon polaritons launched by an Ag antenna reveals V-shaped fringes, a phenomenon widely reported in previous studies (*8, 9, 14, 16–18*). This pattern differs markedly from the simulated $|E_z|$ distribution (Fig.S9d) that reflects the intrinsic directional energy flow. The discrepancy arises from the detection mechanism: the s-SNOM signal combines the propagating polariton response (sP) with the much stronger

local tip-induced response (sL), such that the recorded signal is $s_3 = |sP + sL|$ (*64, 65*). The dominance of the local response is evident in Fig.S9a, where sL corresponds to regions without polariton fringes and reaches values around 10, comparable to the total near-field amplitude of 9~11. Simulations (Fig.S9e) incorporating a constant background ($sL \approx 10$, relative to $|E_z| \sim 1$ in Fig.S9d) successfully reproduce the experimental observations. In this case, the resulting patterns, shaped by constructive and destructive interference, resemble the wavefronts of $\mathrm{Re}(E_z)$ (Fig.S9c), confirming that the local tip response effectively obscures directional propagation signatures.

As summarized in Fig.S9b, the directional amplitude peaks evident in $|E_z|$ are submerged by the dominant sL background, and thus remain barely discernible above the experimental noise. This limitation suggests that further advances in near-field detection are required, particularly techniques that suppress the local tip response. A promising candidate is near-field photocurrent mapping (*66–70*), where polariton waves launched by the metallic tip generate an in-plane photocurrent in suitable optoelectronic materials, providing a direct probe of the polariton field without contamination from the local response.

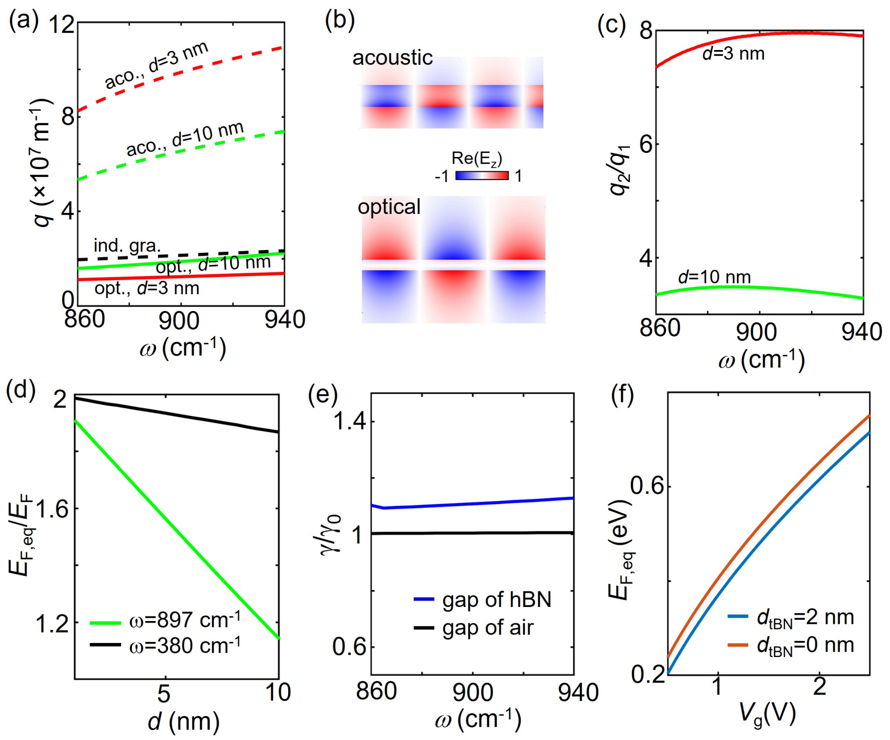

Figure S4: Further analysis of the equivalent model. (a) Plasmon dispersions for the individual graphene (black dashed), and the acoustic and optical modes for hBN thickness of d=3 nm (red) and 10 nm (green), respectively. E_F=0.2 eV here. (b) Spatial distributions of Re(E_z) for the acoustic and optical plasmon modes. Here, the spatial size is not to be scaled. (c) Ratio of polariton momenta for acoustic (q_2) and optical (q_1) modes at interlayer spacings d=3 and 10 nm. (d) Ratio of equivalent Fermi level $E_{F,eq}$ to E_F as a function of interlayer spacing for light frequencies of 897 and 380 cm^{-1}. (e) Ratio of optical mode damping rate γ to that of individual graphene (γ_0) for hBN (blue) and air (black) interlayer spacers. d =3 nm and E_F=0.3 eV. (f) Influence of the top hBN layer on the equivalent Fermi level for the experimental sample shown in Fig.3 of the main text.

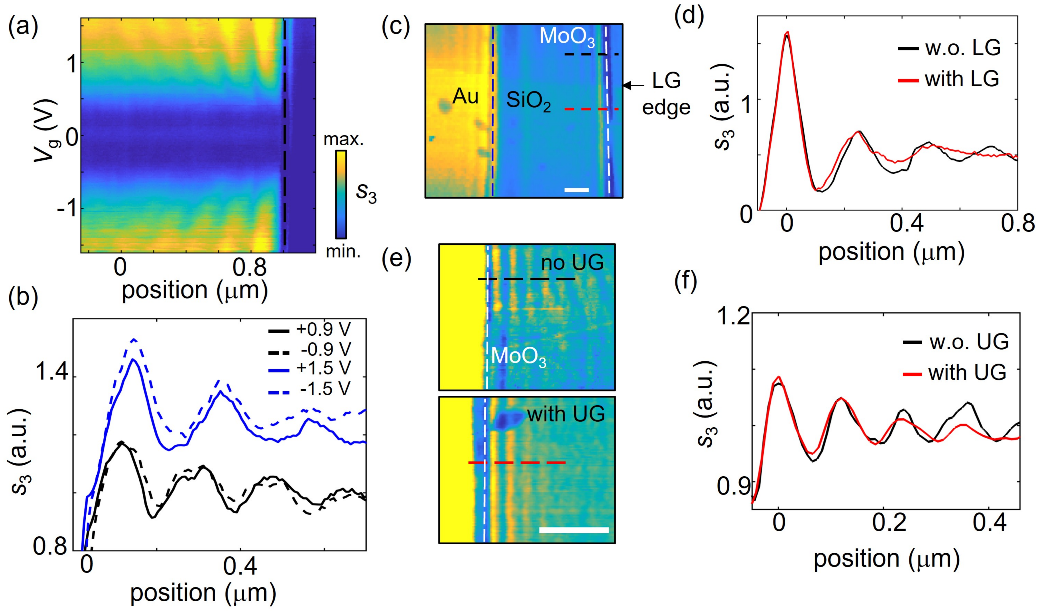

Figure S5: Initial doping of the UG and LG. (a) s_3 profiles across the UG edge (along the green dashed line in Fig.3c of the main text) with V_g swept from 1.6 to -1.6 V. (b) Extracted profiles at $V_g = \pm 0.9$ and 1.5 V from (a), highlighting the symmetric variation around zero bias. (c) Near-field amplitude (s_3) image near the LG edge in the absence of UG. The white dashed line marks the MoO_3 edge, and the green dashed line indicates the boundary between the Au substrate (left) and SiO_2 (right). The black arrow highlights the LG edge, separating regions with (below, higher s_3) and without (above, lower s_3) the LG. (d) Profiles extracted along the black (without LG) and red (with LG) lines in (c). (e,f) Same as (c,d), but for regions with and without the UG in the absence of the LG. Scale bars, 0.5 μm.

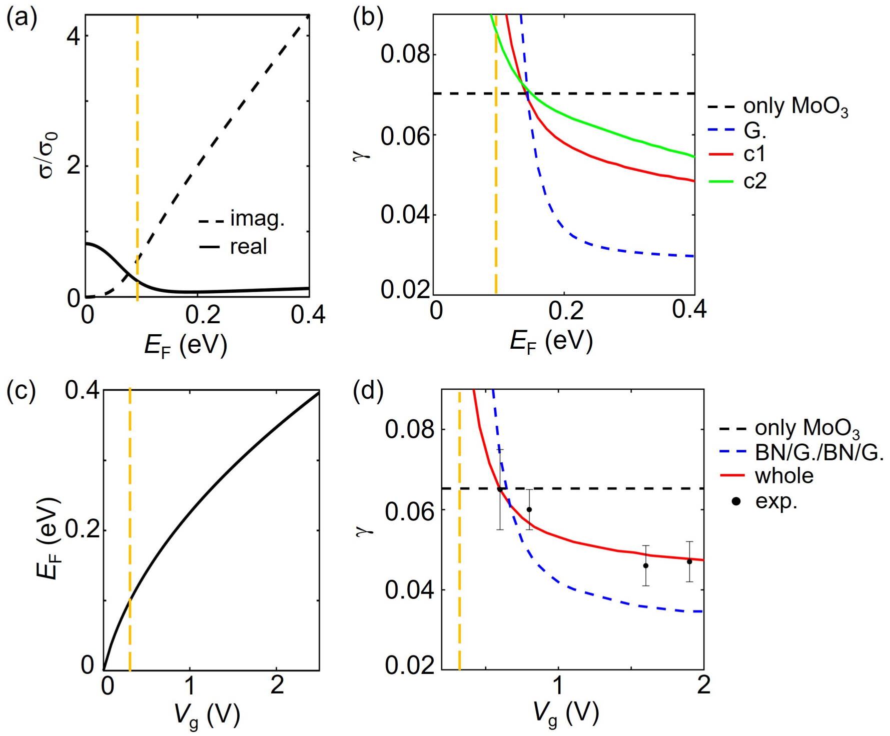

Figure S6: Analysis of polariton damping rates. (a) Optical conductivity of graphene as a function of E_F. Scattering time of $\tau = 0.2$ ps is taken. (b) Damping rates for MoO_3 v-HPhPs (black dashed), individual graphene plasmons (blue dashed), and the graphene–MoO_3 heterostructure for cases c1 and c2 in Fig.1 of the main text. (c) Dependence of the graphene Fermi level E_F on interlayer voltage V_g for the experimental configuration in Fig.3 of the main text. (d) Corresponding damping rates. Yellow dashed lines in panels (a–d) indicate $E_F = 0.1$ eV. The light frequency is 931cm^{-1}.

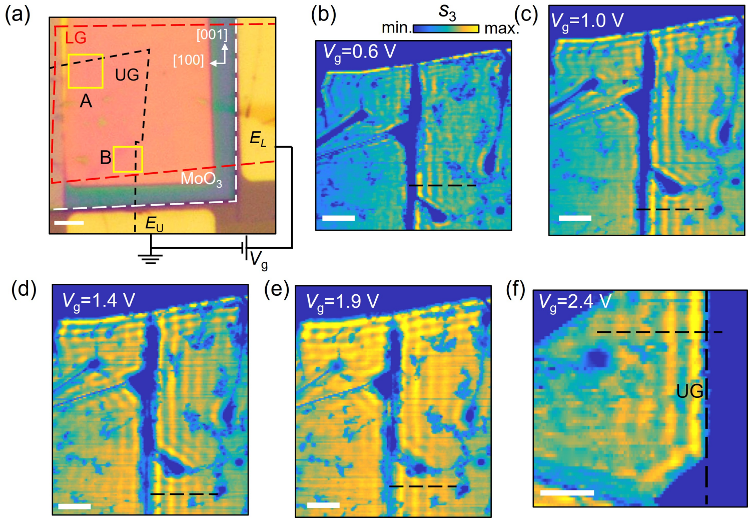

Figure S7: Additional experimental results during V_g sweeping. (a) Optical micrograph of the sample investigated in Fig.3 of the main text, with regions A and B highlighted in yellow. (b–e) Near-field amplitude (s_3) images measured in region A for V_g ranging from 0.6 to 1.9 V. (f) s_3 image of region B at V_g = 2.4 V. Scale bars: 3μm in (a) and 0.5 μm in (b–f).

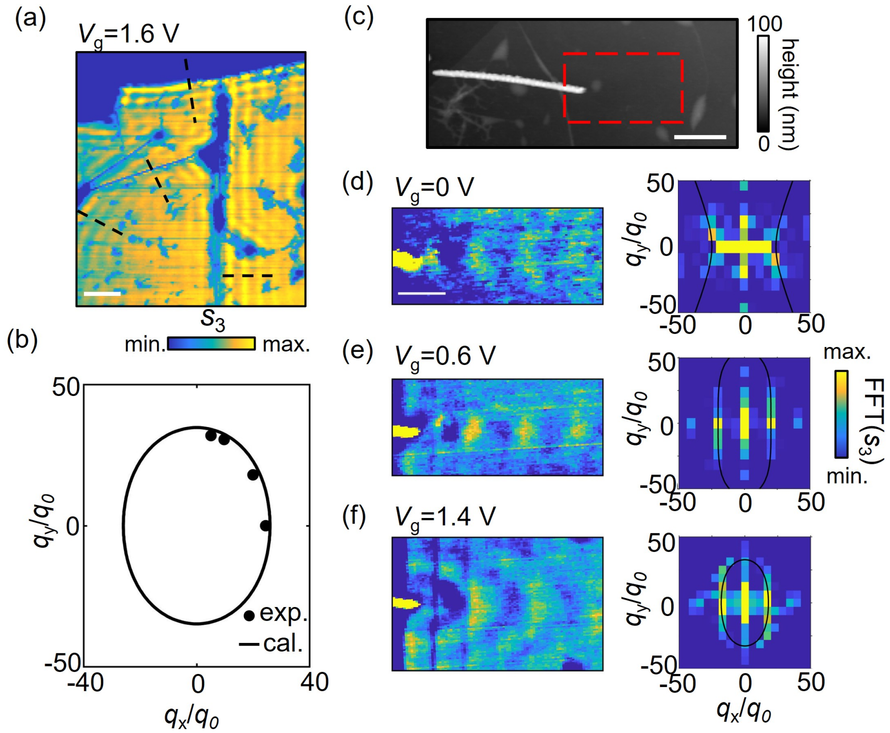

Figure S8: Extra experimental data for IFCs and polariton wavefronts. (a) Near-field s_3 image of region A in Fig.S7a measured at V_g=1.6 V. (b) Comparison between experimentally extracted data (dots, along the dashed lines in (a)) and the calculated IFC for $E_{F,eq}$=0.53 eV. (c) AFM image of the sample shown in Fig.4f of the main text, with an Ag antenna (3 μm length × 150 nm width × 80 nm height) placed on the heterostructure. (d–f) Spatial near-field images (left) recorded within the red dashed region of (c) and their corresponding Fourier transform images (right). The black solid curves in the right panels represent theoretical calculations. Scale bars: 500 nm (a, d–f) and 1 μm (c).

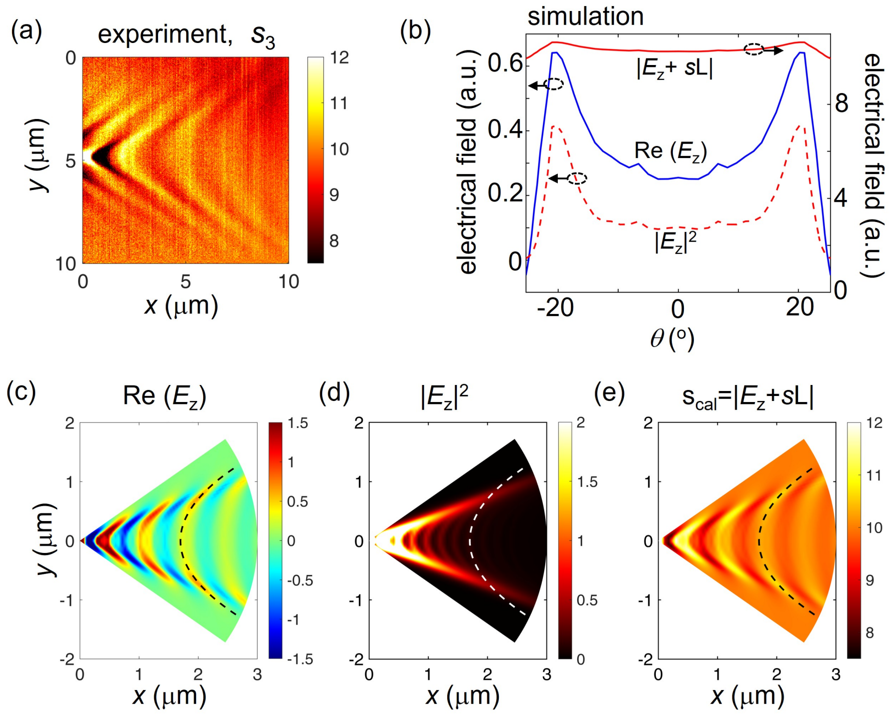

Figure S9: Analyze the influence of the local near-field response of the s-SNOM tip on the observation of directional energy transport. (a) Representative near-field optical amplitude s_3 image of polariton wavefronts in α-MoO_3/SiO_2, launched by an Ag antenna under excitation at 897 cm^{-1}. (b) Simulated angular profiles extracted along the dashed curves in (c–e), plotted relative to the source position. (c, d) Calculated spatial distributions of Re(E_z) (c) and $|E_z|^2$ (d, same as Fig.5a in the main text), excited by a vertically polarized point dipole at position [0,0]. (e) Spatial distribution of $|E_z+sL|$, with sL=10 introduced to mimic the local near-field response of the s-SNOM tip.

REFERENCES AND NOTES

1. S. Dai, Z. Fei, Q. Ma, A. S. Rodin, M. Wagner, A. S. McLeod, M. K. Liu, W. Gannett, W. Regan, K. Watanabe, T. Taniguchi, M. Thiemens, G. Domínguez, A. H. Castro Neto, A. Zettl, F. Keilmann, P. Jarillo-Herrero, M. M. Fogler, D. N. Basov, Tunable phonon polaritons in atomically thin van der Waals crystals of boron nitride. *Science* **343**, 1125–1129 (2014).

2. J. D. Caldwell, L. Lindsay, V. Giannini, I. Vurgaftman, T. L. Reinecke, S. A. Maier, O. J. Glembocki, Low-loss, infrared and terahertz nanophotonics using surface phonon polaritons. *Nanophotonics* **4**, 44–68 (2015).

3. D. N. Basov, M. M. Fogler, F. J. García de Abajo, Polaritons in van der Waals materials. *Science* **354**, aag1992 (2016).

4. Y. Wu, J. Duan, W. Ma, Q. Ou, P. Li, P. Alonso-González, J. D. Caldwell, Q. Bao, Manipulating polaritons at the extreme scale in van der Waals materials. *Nat. Rev. Phys.* **4**, 578–594 (2022).

5. H. Wang, A. Kumar, S. Dai, X. Lin, Z. Jacob, S.-H. Oh, V. Menon, E. Narimanov, Y. D. Kim, J.-P. Wang, P. Avouris, L. M. Moreno, J. Caldwell, T. Low, Planar hyperbolic polaritons in 2D van der Waals materials. *Nat. Commun.* **15**, 69 (2024).

6. E. Galiffi, G. Carini, X. Ni, G. Álvarez-Pérez, S. Yves, E. M. Renzi, R. Nolen, S. Wasserroth, M. Wolf, P. Alonso-Gonzalez, A. Paarmann, A. Alù, Extreme light confinement and control in low-symmetry phonon-polaritonic crystals. *Nat. Rev. Mater.* **9**, 9–28 (2024).

7. J. D. Caldwell, A. V. Kretinin, Y. Chen, V. Giannini, M. M. Fogler, Y. Francescato, C. T. Ellis, J. G. Tischler, C. R. Woods, A. J. Giles, M. Hong, K. Watanabe, T. Taniguchi, S. A. Maier, K. S. Novoselov, Sub-diffractional volume-confined polaritons in the natural hyperbolic material hexagonal boron nitride. *Nat. Commun.* **5**, 5221 (2014).

8. W. Ma, P. Alonso-González, S. Li, A. Y. Nikitin, J. Yuan, J. Martín-Sánchez, J. Taboada-Gutiérrez, I. Amenabar, P. Li, S. Vélez, C. Tollan, Z. Dai, Y. Zhang, S. Sriram, K. Kalantar-Zadeh, S.-T. Lee, R. Hillenbrand, Q. Bao, In-plane anisotropic and ultra-low-loss polaritons in a natural van der waals crystal. *Nature* **562**, 557–562 (2018).

9. Z. Zheng, N. Xu, S. L. Oscurato, M. Tamagnone, F. Sun, Y. Jiang, Y. Ke, J. Chen, W. Huang, W. L. Wilson, A. Ambrosio, S. Deng, H. Chen, A mid-infrared biaxial hyperbolic van der Waals crystal. *Sci. Adv.* **5**, eaav8690 (2019).

10. L. Liu, L. Xiong, C. Wang, Y. Bai, W. Ma, Y. Wang, P. Li, G. Li, Q. J. Wang, F. J. Garcia-Vidal, Z. Dai, G. Hu, Long-range hyperbolic polaritons on a non-hyperbolic crystal surface. *Nature* **644**, 76–82 (2025).

11. W. Ma, G. Hu, D. Hu, R. Chen, T. Sun, X. Zhang, Q. Dai, Y. Zeng, A. Alù, C.-W. Qiu, P. Li, Ghost hyperbolic surface polaritons in bulk anisotropic crystals. *Nature* **596**, 362–366 (2021).

12. N. C. Passler, X. Ni, G. Hu, J. R. Matson, G. Carini, M. Wolf, M. Schubert, A. Alù, J. D. Caldwell, T. G. Folland, A. Paarmann, Hyperbolic shear polaritons in low-symmetry crystals. *Nature* **602**, 595–600 (2022).

13. S. Dai, Q. Ma, T. Andersen, A. S. McLeod, Z. Fei, M. K. Liu, M. Wagner, K. Watanabe, T. Taniguchi, M. Thiemens, F. Keilmann, P. Jarillo-Herrero, M. M. Fogler, D. N. Basov, Subdiffractional focusing and guiding of polaritonic rays in a natural hyperbolic material. *Nat. Commun.* **6**, 6963 (2015).

14. G. Hu, Q. Ou, G. Si, Y. Wu, J. Wu, Z. Dai, A. Krasnok, Y. Mazor, Q. Zhang, Q. Bao, C.-W. Qiu, A. Alù, Topological polaritons and photonic magic angles in twisted α-MoO_3 bilayers. *Nature* **582**, 209–213 (2020).

15. Z. Zheng, F. Sun, W. Huang, J. Jiang, R. Zhan, Y. Ke, H. Chen, S. Deng, Phonon polaritons in twisted double-layers of hyperbolic van der Waals crystals. *Nano Lett.* **20**, 5301–5308 (2020).

16. M. Chen, X. Lin, T. H. Dinh, Z. Zheng, J. Shen, Q. Ma, H. Chen, P. Jarillo-Herrero, S. Dai, Configurable phonon polaritons in twisted α-moo3. *Nat. Mater.* **19**, 1307–1311 (2020).

17. J. Duan, N. Capote-Robayna, J. Taboada-Gutiérrez, G. Álvarez-Pérez, I. Prieto, J. Martín-Sánchez, A. Y. Nikitin, P. Alonso-González, Twisted nano-optics: Manipulating light at the nanoscale with twisted phonon polaritonic slabs. *Nano Lett.* **20**, 5323–5329 (2020).

18. J. Duan, G. Álvarez-Pérez, C. Lanza, K. Voronin, A. I. F. Tresguerres-Mata, N. Capote-Robayna, J. Álvarez-Cuervo, A. T. Martín-Luengo, J. Martín-Sánchez, V. S. Volkov, A. Y. Nikitin, P. Alonso-González, Multiple and spectrally robust photonic magic angles in reconfigurable α-MoO_3 trilayers. *Nat. Mater.* **22**, 867–872 (2023).

19. J. Duan, G. Álvarez-Pérez, A. I. F. Tresguerres-Mata, J. Taboada-Gutiérrez, K. V. Voronin, A. Bylinkin, B. Chang, S. Xiao, S. Liu, J. H. Edgar, J. I. Martín, V. S. Volkov, R. Hillenbrand, J. Martín-Sánchez, A. Y. Nikitin, P. Alonso-González, Planar refraction and lensing of highly confined polaritons in anisotropic media. *Nat. Commun.* **12**, 4325 (2021).

20. G. Álvarez-Pérez, J. Duan, J. Taboada-Gutiérrez, Q. Ou, E. Nikulina, S. Liu, J. H. Edgar, Q. Bao, V. Giannini, R. Hillenbrand, J. Martín-Sánchez, A. Y. Nikitin, P. Alonso-González, Negative reflection of nanoscale-confined polaritons in a low-loss natural medium. *Sci. Adv.* **8**, eabp8486 (2022).

21. A. J. Sternbach, S. L. Moore, A. Rikhter, S. Zhang, R. Jing, Y. Shao, B. S. Y. Kim, S. Xu, S. Liu, J. H. Edgar, A. Rubio, C. Dean, J. Hone, M. M. Fogler, D. N. Basov, Negative refraction in hyperbolic hetero-bicrystals. *Science* **379**, 555–557 (2023).

22. H. Hu, N. Chen, H. Teng, R. Yu, M. Xue, K. Chen, Y. Xiao, Y. Qu, D. Hu, J. Chen, Z. Sun, P. Li, F. J. García de Abajo, Q. Dai, Gate-tunable negative refraction of mid-infrared polaritons. *Science* **379**, 558–561 (2023).

23. A. Bylinkin, M. Schnell, M. Autore, F. Calavalle, P. Li, J. Taboada-Gutierrez, S. Liu, J. H. Edgar, F. Casanova, L. E. Hueso, P. Alonso-González, A. Y. Nikitin, R. Hillenbrand, Real-space observation of vibrational strong coupling between propagating phonon polaritons and organic molecules. *Nat. Photonics* **15**, 197–202 (2021).

24. H. H. Sheinfux, L. Orsini, M. Jung, I. Torre, M. Ceccanti, S. Marconi, R. Maniyara, D. B. Ruiz, A. Hötger, R. Bertini, S. Castilla, N. C. H. Hesp, E. Janzen, A. Holleitner, V. Pruneri, J. H. Edgar, G. Shvets, F. H. L. Koppens, High-quality nanocavities through multimodal confinement of hyperbolic polaritons in hexagonal boron nitride. *Nat. Mater.* **23**, 499–505 (2024).

25. P. Li, M. Lewin, A. V. Kretinin, J. D. Caldwell, K. S. Novoselov, T. Taniguchi, K. Watanabe, F. Gaussmann, T. Taubner, Hyperbolic phonon-polaritons in boron nitride for near-field optical imaging and focusing. *Nat. Commun.* **6**, 7507 (2015).

26. J. Duan, A. T. Martín-Luengo, C. Lanza, S. Partel, K. Voronin, A. I. F. Tresguerres-Mata, G. Álvarez-Pérez, A. Y. Nikitin, J. Martín-Sánchez, P. Alonso-González, Canalization-based super-resolution imaging using an individual van der Waals thin layer. *Sci. Adv.* **11**, eads0569 (2025).

27. Q. Ou, S. Xue, W. Ma, J. Yang, G. Si, L. Liu, G. Zhong, J. Liu, Z. Xie, Y. Xiao, T. Sun, D. Yuan, K. Kalantar-Zadeh, P. Li, Z. Dai, H. Chen, Q. Bao, Natural van der Waals canalization lens for non-destructive nanoelectronic circuit imaging and inspection. *Adv. Mater.* **37**, e2504526 (2025).

28. S. Dai, Q. Ma, M. K. Liu, T. Andersen, Z. Fei, M. D. Goldflam, M. Wagner, K. Watanabe, T. Taniguchi, M. Thiemens, F. Keilmann, G. C. A. M. Janssen, S.-E. Zhu, P. Jarillo-Herrero, M. M. Fogler, D. N. Basov, Graphene on hexagonal boron nitride as a tunable hyperbolic metamaterial. *Nat. Nanotechnol.* **10**, 682–686 (2015).

29. F. L. Ruta, B. S. Y. Kim, Z. Sun, D. J. Rizzo, A. S. McLeod, A. Rajendran, S. Liu, A. J. Millis, J. C. Hone, D. N. Basov, Surface plasmons induce topological transition in graphene/α-MoO_3 heterostructures. *Nat. Commun.* **13**, 3719 (2022).

30. H. Hu, N. Chen, H. Teng, R. Yu, Y. Qu, J. Sun, M. Xue, D. Hu, B. Wu, C. Li, J. Chen, M. Liu, Z. Sun, Y. Liu, P. Li, S. Fan, F. J. García de Abajo, Q. Dai, Doping-driven topological polaritons in graphene/α-MoO_3 heterostructures. *Nat. Nanotechnol.* **17**, 940–946 (2022).

31. Y. Zeng, Q. Ou, L. Liu, C. Zheng, Z. Wang, Y. Gong, X. Liang, Y. Zhang, G. Hu, Z. Yang, C.-W. Qiu, Q. Bao, H. Chen, Z. Dai, Tailoring topological transitions of anisotropic polaritons by interface engineering in biaxial crystals. *Nano Lett.* **22**, 4260–4268 (2022).

32. G. Álvarez-Pérez, A. González-Morán, N. Capote-Robayna, K. V. Voronin, J. Duan, V. S. Volkov, P. Alonso-González, A. Y. Nikitin, Active tuning of highly anisotropic phonon polaritons in van der Waals crystal slabs by gated graphene. *ACS Photonics* **9**, 383–390 (2022).

33. A. Bapat, S. Dixit, Y. Gupta, T. Low, A. Kumar, Gate tunable light–matter interaction in natural biaxial hyperbolic van der Waals heterostructures. *Nanophotonics* **11**, 2329–2340 (2022).

34. Z. Zhou, R. Song, X. Junbo, X. Ni, Z. Dang, Z. Zhao, J. Quan, S. Dong, H. Weida, D. Huang, K. Chen, Z. Wang, X. Cheng, M. B. Raschke, A. Alù, T. Jiang, Gate-tuning hybrid polaritons in twisted α-MoO_3/graphene heterostructures. *Nano Lett.* **23**, 11252–11259 (2023).

35. J. Duan, F. J. Alfaro-Mozaz, J. Taboada-Gutiérrez, I. Dolado, G. Álvarez-Pérez, E. Titova, A. Bylinkin, A. I. F. Tresguerres-Mata, J. Martín-Sánchez, S. Liu, J. H. Edgar, D. A. Bandurin, P. Jarillo-Herrero, R. Hillenbrand, A. Y. Nikitin, P. Alonso-González, Active and passive tuning of ultranarrow resonances in polaritonic nanoantennas. *Adv. Mater.* **34**, e2104954 (2022).

36. S. Castilla, H. Agarwal, I. Vangelidis, Y. V. Bludov, D. A. Iranzo, A. Grabulosa, M. Ceccanti, M. I. Vasilevskiy, R. K. Kumar, E. Janzen, J. H. Edgar, K. Watanabe, T. Taniguchi, N. M. R. Peres, E. Lidorikis, F. H. L. Koppens, Electrical spectroscopy of polaritonic nanoresonators. *Nat. Commun.* **15**, 8635 (2024).

37. D. J. Rizzo, B. S. Jessen, Z. Sun, F. L. Ruta, J. Zhang, J.-Q. Yan, L. Xian, A. S. McLeod, M. E. Berkowitz, K. Watanabe, T. Taniguchi, S. E. Nagler, D. G. Mandrus, A. Rubio, M. M. Fogler, A. J. Millis, J. C. Hone, C. R. Dean, D. N. Basov, Charge-transfer plasmon polaritons at graphene/α-$RuCl_3$ interfaces. *Nano Lett.* **20**, 8438–8445 (2020).

38. B. S. Y. Kim, A. J. Sternbach, M. S. Choi, Z. Sun, F. L. Ruta, Y. Shao, A. S. McLeod, L. Xiong, Y. Dong, T. S. Chung, A. Rajendran, S. Liu, A. Nipane, S. H. Chae, A. Zangiabadi, X. Xu, A. J. Millis, P. J. Schuck, C. R. Dean, J. C. Hone, D. N. Basov, Ambipolar charge-transfer graphene plasmonic cavities. *Nat. Mater.* **22**, 838–843 (2023).

39. A. Woessner, M. B. Lundeberg, Y. Gao, A. Principi, P. Alonso-González, M. Carrega, K. Watanabe, T. Taniguchi, G. Vignale, M. Polini, J. Hone, R. Hillenbrand, F. H. L. Koppens, Highly confined low-loss plasmons in graphene–boron nitride heterostructures. *Nat. Mater.* **14**, 421–425 (2015).

40. G. X. Ni, A. S. McLeod, Z. Sun, L. Wang, L. Xiong, K. W. Post, S. S. Sunku, B.-Y. Jiang, J. Hone, C. R. Dean, M. M. Fogler, D. N. Basov, Fundamental limits to graphene plasmonics. *Nature* **557**, 530–533 (2018).

41. W. Luo, A. B. Kuzmenko, J. Qi, N. Zhang, W. Wei, M. Ren, X. Zhang, W. Cai, J. Xu, Nanoinfrared characterization of bilayer graphene conductivity under dual-gate tuning. *Nano Lett.* **21**, 5151–5157 (2021).

42. A. Woessner, A. Misra, Y. Cao, I. Torre, A. Mishchenko, M. B. Lundeberg, K. Watanabe, T. Taniguchi, M. Polini, K. S. Novoselov, F. H. L. Koppens, Propagating plasmons in a charge-neutral quantum tunneling transistor. *ACS Photonics* **4**, 3012–3017 (2017).

43. R. E. V. Profumo, R. Asgari, M. Polini, A. H. MacDonald, Double-layer graphene and topological insulator thin-film plasmons. *Phys. Rev. B* **85**, 085443 (2012).

44. C. Hu, A. Deng, P. Shen, L. Xingdong, X. Zhou, T. Wu, X. Huang, Y. Dong, K. Watanabe, T. Taniguchi, G. Xie, X. Li, Q. Liang, Z. Shi, Direct imaging of interlayer-coupled symmetric and antisymmetric plasmon modes in graphene/hbn/graphene heterostructures. *Nanoscale* **13**, 14628–14635 (2021).

45. J. Duan, G. Álvarez-Pérez, K. V. Voronin, I. Prieto, J. Taboada-Gutiérrez, V. S. Volkov, J. Martín-Sánchez, A. Y. Nikitin, P. Alonso-González, Enabling propagation of anisotropic polaritons along forbidden directions via a topological transition. *Sci. Adv.* **7**, eabf2690 (2021).

46. C. Hu, T. Sun, Y. Zeng, W. Ma, Z. Dai, X. Yang, X. Zhang, P. Li, Source-configured symmetry-broken hyperbolic polaritons. *eLight* **3**, 14 (2023).

47. J. Álvarez-Cuervo, M. Obst, S. Dixit, G. Carini, A. I. F. Tresguerres-Mata, C. Lanza, E. Terán-García, G. Álvarez-Pérez, L. F. Álvarez-Tomillo, K. Diaz-Granados, R. Kowalski, A. S. Senerath, N. S. Mueller, L. Herrer, J. M. De Teresa, S. Wasserroth, J. M. Klopf, T. Beechem, M. Wolf, L. M. Eng, T. G. Folland, A. Tarazaga Martín-Luengo, J. Martín-Sánchez, S. C. Kehr, A. Y. Nikitin, J. D. Caldwell, P. Alonso-González, A. Paarmann, Unidirectional ray polaritons in twisted asymmetric stacks. *Nat. Commun.* **15**, 9042 (2024).

48. Q. Zhang, G. Hu, W. Ma, P. Li, A. Krasnok, R. Hillenbrand, A. Alù, C.-W. Qiu, Interface nano-optics with van der Waals polaritons. *Nature* **597**, 187–195 (2021).

49. W. Hutchins, S. Zare, D. M. Hirt, J. A. Tomko, J. R. Matson, K. Diaz-Granados, M. Long III, M. He, T. Pfeifer, J. Li, J. H. Edgar, J.-P. Maria, J. D. Caldwell, P. E. Hopkins, Ultrafast evanescent heat transfer across solid interfaces via hyperbolic phonon–polariton modes in hexagonal boron nitride. *Nat. Mater.* **24**, 698–706 (2025).

50. S. Xue, L. Liu, X. Huang, Y. Li, Y. Xiao, S. Lu, P. Li, Z. Dai, H. Chen, Asymmetric polaritons with half-hyperbolic shaped propagation. *Nano Lett.* **25**, 8923–8930 (2025).

51. T. Sun, R. Chen, W. Ma, H. Wang, Q. Yan, J. Luo, S. Zhao, X. Zhang, P. Li, Van der waals quaternary oxides for tunable low-loss anisotropic polaritonics. *Nat. Nanotechnol.* **19**, 758–765 (2024).

52. N. Chen, H. Teng, H. Hai, M. Liu, C. Jiang, Z. Xue, H. Zhu, J. Gui, P. Li, A. Alù, Q. Dai, Flatland wakes based on leaky hyperbolic polaritons. *Nat. Mater.* **24**, 1569–1575 (2025).

53. H. Teng, N. Chen, H. Hai, F. J. García de Abajo, Q. Dai, Steering and cloaking of hyperbolic polaritons at deep-subwavelength scales. *Nat. Commun.* **15**, 4463 (2024).

54. N. Ocelic, A. Huber, R. Hillenbrand, Pseudoheterodyne detection for background-free near-field spectroscopy. *Appl. Phys. Lett.* **89**, 101124 (2006).

55. G. Álvarez-Pérez, K. V. Voronin, V. S. Volkov, P. Alonso-González, A. Y. Nikitin, Analytical approximations for the dispersion of electromagnetic modes in slabs of biaxial crystals. *Phys. Rev. B* **100**, 235408 (2019).

56. L. A. Falkovsky, Optical properties of graphene and IV–VI semiconductors. *Phys. Usp.* **51**, 887–897 (2008).

57. M. B. Lundeberg, Y. Gao, R. Asgari, C. Tan, B. Van Duppen, M. Autore, P. Alonso-González, A. Woessner, K. Watanabe, T. Taniguchi, R. Hillenbrand, J. Hone, M. Polini, F. H. L. Koppens, Tuning quantum nonlocal effects in graphene plasmonics. *Science* **357**, 187–191 (2017).

58. T. V. A. G. de Oliveira, T. Nörenberg, G. Álvarez-Pérez, L. Wehmeier, J. Taboada-Gutiérrez, M. Obst, F. Hempel, E. J. H. Lee, J. M. Klopf, I. Errea, A. Y. Nikitin, S. C. Kehr, P. Alonso-González, L. M. Eng, Nanoscale-confined terahertz polaritons in a van der Waals crystal. *Adv. Mater.* **33**, e2005777 (2021).

59. S. Chen, A. Bylinkin, Z. Wang, M. Schnell, G. Chandan, P. Li, A. Y. Nikitin, S. Law, R. Hillenbrand, Real-space nanoimaging of THz polaritons in the topological insulator Bi_2Se_3. *Nat. Commun.* **13**, 1374 (2022).

60. Y. Luan, J. Qian, M. Kim, K.-M. Ho, Y. Shi, Y. Li, C.-Z. Wang, M. C. Tringides, Z. Fei, Imaging stacking-dependent surface plasmon polaritons in trilayer graphene. *Phys. Rev. Applied* **18**, 024052 (2022).

61. G. L. Yu, R. Jalil, B. Belle, A. S. Mayorov, P. Blake, F. Schedin, S. V. Morozov, L. A. Ponomarenko, F. Chiappini, S. Wiedmann, U. Zeitler, M. I. Katsnelson, A. K. Geim, K. S. Novoselov, D. C. Elias, Interaction phenomena in graphene seen through quantum capacitance. *Proc. Natl. Acad. Sci. U.S.A.* **110**, 3282–3286 (2013).

62. N. C. H. Hesp, I. Torre, D. Rodan-Legrain, P. Novelli, Y. Cao, S. Carr, S. Fang, P. Stepanov, D. Barcons-Ruiz, H. H. Sheinfux, K. Watanabe, T. Taniguchi, D. K. Efetov, E. Kaxiras, P. Jarillo-Herrero, M. Polini, F. H. L. Koppens, Observation of interband collective excitations in twisted bilayer graphene. *Nat. Phys.* **17**, 1162–1168 (2021).

63. L. Novotny, B. Hecht, *Principles of nano-optics* (Cambridge Univ. Press, 2012).

64. A. Y. Pablo Alonso-González, F. G. Nikitin, A. Centeno, A. Pesquera, S. Vélez, J. Chen, G. Navickaite, F. Koppens, A. Zurutuza, A. Zurutuza, F. Casanova, L. E. Hueso, R. Hillenbrand, Controlling graphene plasmons with resonant metal antennas and spatial conductivity patterns. *Science* **344**, 1369–1373 (2014).

65. P. Li, I. Dolado, F. J. Alfaro-Mozaz, F. Casanova, L. E. Hueso, S. Liu, J. H. Edgar, A. Y. Nikitin, S. Vélez, R. Hillenbrand, Infrared hyperbolic metasurface based on nanostructured van der Waals materials. *Science* **359**, 892–896 (2018).

66. A. Woessner, P. Alonso-González, M. B. Lundeberg, Y. Gao, J. E. Barrios-Vargas, G. Navickaite, Q. Ma, D. Janner, K. Watanabe, A. W. Cummings, T. Taniguchi, V. Pruneri, S. Roche, P. Jarillo-Herrero, J. Hone, R. Hillenbrand, F. H. L. Koppens, Near-field photocurrent nanoscopy on bare and encapsulated graphene. *Nat. Commun.* **7**, 10783 (2016).

67. M. B. Lundeberg, Y. Gao, A. Woessner, C. Tan, P. Alonso-González, K. Watanabe, T. Taniguchi, J. Hone, R. Hillenbrand, F. H. L. Koppens, Thermoelectric detection and imaging of propagating graphene plasmons. *Nat. Mater.* **16**, 204–207 (2017).

68. P. Alonso-González, A. Y. Nikitin, Y. Gao, A. Woessner, M. B. Lundeberg, A. Principi, N. Forcellini, W. Yan, S. Vélez, A. J. Huber, K. Watanabe, T. Taniguchi, F. Casanova, L. E. Hueso, M. Polini, J. Hone, F. H. L. Koppens, R. Hillenbrand, Acoustic terahertz graphene plasmons revealed by photocurrent nanoscopy. *Nat. Nanotechnol.* **12**, 31–35 (2017).

69. S. S. Sunku, D. Halbertal, T. Stauber, S. Chen, A. S. McLeod, A. Rikhter, M. E. Berkowitz, C. F. B. Lo, D. E. Gonzalez-Acevedo, J. C. Hone, C. R. Dean, M. M. Fogler, D. N. Basov, Hyperbolic enhancement of photocurrent patterns in minimally twisted bilayer graphene. *Nat. Commun.* **12**, 1641 (2021).

70. N. C. H. Hesp, I. Torre, D. Barcons-Ruiz, H. H. Sheinfux, K. Watanabe, T. Taniguchi, R. K. Kumar, F. H. L. Koppens, Nano-imaging photoresponse in a moiré unit cell of minimally twisted bilayer graphene. *Nat. Commun.* **12**, 1640 (2021).
